# Three-dimensional simulation of clouds of multi-disperse evaporating saliva droplets in a train cabin

**DOI:** 10.1063/5.0059649

**Published:** 2021-08-12

**Authors:** M. Visone, M. Lanzetta, M. Lappa, C. Lanzaro, L. Polizio

**Affiliations:** 1 Blue Engineering, Via Albenga 98, Rivoli, Turin 10098, Italy; 2 University of Strathclyde, James Weir Building, 75 Montrose Street, Glasgow G1 1XJ, United Kingdom

## Abstract

In line with recent ongoing efforts to collect crucial information about the mechanisms of virus diffusion and put them in relation to the effective complexity of the several natural or artificial environments where human beings leave and operate, the present study deals with the dispersion of evaporating saliva droplets in the cabin of an interregional train. A relevant physical model is constructed taking into account the state of the art in terms of existing paradigms and their ability to represent some fundamental aspects related to the evolution in time of a cloud of multi-disperse droplets. Conveniently, such a theoretical framework is turned into a computational one that relies on low Mach-number asymptotics and can therefore take advantage of the typical benefits (relatively low computational cost) associated with pressure-based methods. Numerical simulations are used to predict the flow established in the cabin as a result of the ventilation systems and related settings dictated by considerations on passenger comfort. The solution of two-way coupled Lagrangian evolution equations is used to capture the associated dynamics of the dispersed phase and predict its transport in conjunction with the peculiar topology of the considered flow and morphology of solid surfaces, which bound it (including the human beings). Typical physiological processes such as talking or coughing are considered. An analysis on the impact of the multiplicity of droplet sources is also conducted, thereby providing some indications in terms of potential risks for the cabin occupants.

## I. INTRODUCTION

Since the beginning of the COVID-19 pandemic (World Health Organization^1^), CFD (computational fluid dynamics) has enjoyed a widespread use for the investigation of the inherent droplet-based mechanisms by which the virus infection can be propagated. Such a practice has flourished due to the intrinsic ability of this branch of computational physics to complement experimental and theoretical fluid dynamics by providing an alternative (extremely effective) means of simulating real processes for conditions often unavailable experimentally or not practically realizable. The intrinsic reasons of such a modus operandi (and its success) can also be directly rooted in the decision of several research groups (having different backgrounds and perspectives) to move from their traditional heartlands of applied engineering or fundamental research to new lines of inquiry aimed at a better understanding of the mechanisms of virus diffusion.

The resulting articulated numerical studies have provided disjointed glimpses of a wide variety of qualitatively and quantitatively different results in widely different parts of parameter space, essentially reflecting current interpretations or beliefs about the possible cause-and-effect relationships driving the pandemic (Mittal *et al.*^2^). This peculiar endeavor or framework has been supported by the widespread consensus that the transmission of COVID-19 occurs essentially via virus-laden “droplets,” which originate from the respiratory tract of an infected person and are expelled from the mouth and nose in a variety of circumstances. These include anomalous situations such as coughing or sneezing (where droplets can be formed by saliva and by the mucous coating of the lungs and vocal cords, Zhu *et al.*;^3^ Simha and Rao;^4^ Dbouka and Drikakis;^5^ National Research Council;^6^ Fontes *et al.*;^7^ and Li *et al.*^8^) and/or even normal activities such as breathing and talking (Jones and Brosseau;^9^ Asadi *et al.*;^10^ Bourouiba;^11^ CDC;^12^ and Smith *et al.*^13^).

In particular, a first analysis on which many more recent investigations have relied is the original study by Wells,^14,15^ where it was shown that the evolution in time of these droplets is governed by the triadic relationship among inertia, gravity, and evaporation. It is worth highlighting that this realization has naturally led to the introduction of a kind of dichotomy in this category of studies, that is, a net distinction between “large” and “small” droplets; namely, cases where the size of droplets is such that the time they take to settle is smaller than that required for their evaporation, or, vice versa, the sedimentation process is so slow that the liquid part of the droplets is entirely consumed before they reach the ground.

These two paradigms should obviously be regarded as opposite extremes, both being severe approximations to a more complete representation of possible populations of droplets created by human beings. The former may be considered representative of practical circumstances where droplets can actually contaminate surfaces located in a certain neighborhood of the source emitting them; the latter may be used to model situations where the droplets can remain suspended in the air for relatively long times (forming an “aerosol”) and be transported accordingly at large distances from the source (Pendar and Pascoa^16^). The translational relevance or applicability of these realizations to the effective propagation of the infection can be immediately seen by considering that human-to-human transmission of COVID-19 is thought to occur due to some related (concurrent) mechanisms.

These mechanisms are not mutually exclusive, nor are they truly progressive. Relatively large droplets undergoing fast sedimentation can be deposited on surfaces and be transferred according to other human beings coming intentionally or inadvertently in physical contact with such surfaces (Bhardwaj and Agrawal^17,18^). Aerosolized tiny droplets originating from expiratory ejecta can be transported by ambient air currents until they are inhaled by potential recipients.

A careful analysis of the existing literature, however, also indicates that there are reasons to question whether these theories are the only viable contenders for the interpretation or prediction of the infection spreading rate. As an example, the possibility for droplets to reach the nose, mouth, or conjunctiva of another human being should not be regarded as an exclusive prerogative of small droplets. Even large droplets may reach other human beings if expelled with sufficiently high momentum; that is, the “initial conditions” should also be considered as an important aspect of the overall problem (Renzi and Clarke^19^).

In this (already complex *per se*) scenario, evaporation should be seen as an additional influential factor potentially causing the transition from one mechanism to another (as the preferred mode of infection diffusion) depending on environmental conditions. This apparently innocuous observation implies that the problem also strongly depends on the “boundary conditions.” In particular, the rate of evaporation is known to be a function of the droplet surface saturation vapor pressure and the vapor pressure of the surrounding gas, which in turn depends on the degree of humidity (Dbouka and Drikakis^20^ and Li *et al.*^21^). Obviously, the evaporation and movement of the droplets (after being expelled) are predominately dictated by their sizes. The evaporation rate also displays a relationship with the mass-diffusion coefficient, which in turn changes according to the droplet-to-ambient temperature difference and the velocity of the droplet with respect to the surrounding gaseous environment (Xie *et al.*^22^). Winter conditions of low temperature and high relative humidity (RH) can cause more droplets to survive over relatively long times, which may be a possible driver of a second pandemic wave in the autumn and winter seasons (Wang *et al.*^23^).

Superimposed on these aspects is the possibility that droplets are turned with time into small “solid residues.” Indeed, evaporation can cause quick consumption (in a few seconds) of small droplets suspended and transported in air currents, forcing them to form nuclei consisting of virions, salts that were previously dissolved in water (Vejerano and Marr^24^ and Chaudhuri *et al.*^25^), various proteins and pathogens in varying concentrations (Xie *et al.*^22^), and a certain (small) percentage of residual water (Mezhericher *et al.*^26^). These nuclei can span the range from the micrometric to the millimeter size. They can be suspended in the air for hours and are thought to play an important role in the mechanisms of diffusion of the infection as well (van Doremalen *et al.*;^27^ Nicas *et al.*;^28^ and Asadi *et al.*^10^). As explained before, unlike large droplets (which always require a significant amount of initial momentum in order to step away from the source), small droplets and/or related nuclei can cover large distances if properly supported by ambient flows such as those produced in indoor environments (i.e., public buildings, hospitals, homes, offices, classrooms, airplanes, trains, subways, buses) as a result of air conditioning systems and related forced flow (Tang *et al.*;^29^ Zhang and Chen;^30^ Li *et al.*;^31^ Eames *et al.*;^32^ Zhao *et al.*;^33^ Balocco and Liò;^34^ Thatiparti *et al.*;^35^ Yang *et al.*;^36^ Yu *et al.*;^37^ Craven and Settles;^38^ Licina *et al.*;^39^ Abuhegazy *et al.*;^40^ and Zhou and Ji^41^).

At this stage, to put the present work in perspective, we wish to remark that, although an additional theoretical inquiry is needed to elaborate a full accounting of all the basic forces, factors, and physical conditions governing the outbreak diffusion (and efforts are currently in progress along these lines), we now are in a situation where the CFD analysis of all the dynamics illustrated above has reached a sort of maturity, in the sense that the existing numerical techniques are in a position to convey valuable and relevant information concerning most of the questions we may ask on the problem. We wish to highlight as well that, surprisingly, the overwhelming majority of research has focused on relatively simplified configurations where the complexity, intricacy, and tortuosity of the currents transporting droplets have been filtered out in favor of a more fundamental approach (aimed at disentangling the functional relationships governing the underlying dynamics).

A relevant exception is represented by the recent work by Abuhegazy *et al.*^40^ where the location of the “source” has been found to influence strongly the trajectory and deposition distribution of the exhaled aerosol particles and affect the effectiveness of mitigation measures such as glass barriers. According to such a study, particles larger than 20 *μ*m entirely deposit on the ground, desks, and nearby surfaces in the room, while glass barriers can reduce the aerosol transmission of 1 *μ*m particles by ∼92%. Moreover, by opening windows, the particle exit fraction can be increased by ∼38% compared to the case with closed windows.

In the present work, assuming the state of the art in terms of infection transmission models (that is considering a statistical population of droplets that includes both small and large variants and can evaporate), we tackle the problem considering the intricacies, which are typical of an effective (realistic) environment such as that of a train car. Following a common practice in this kind of studies (all based on hybrid Eulerian–Lagrangian techniques, which, therefore, can be said to unify the study of these subjects at least from a numerical point of view), we address the initial boundary value problem (IBVP) numerically solving the classical three-dimensional Navier–Stokes equations for the environment and additional specific equations to track the motion of particles (and their evolution in terms of temperature and mass).

## II. MATHEMATICAL MODEL

As stated in the introduction, we consider evaporating droplets in the air. From a physical point of view, therefore, the problem consists essentially of a multiphase gas (including the air and the water vapor resulting from the saliva evaporation process) and the dispersed liquid droplets. We treat the gaseous phase in the framework of a variable density approach, as discussed in Sec. II A.

### A. Eulerian approach

The governing equations for mass, momentum, and enthalpy in the classical unsteady and compressible (complete) form read

• continuity
  $$\frac{\partial \rho }{\partial t}+\underline{\nabla }\cdot (\rho \underline{V})=0,$$ (1)
• species transport for vapor phase
  $$\frac{\partial (\rho Y_{v})}{\partial t}+\underline{\nabla }\cdot \left(\rho Y_{v}\underline{V}\right)=\underline{\nabla }\cdot \left(\rho D_{v}\underline{\nabla }Y_{v}\right)+S_{v},$$ (2)
• momentum
  $$\frac{\partial }{\partial t}\rho \underline{V}+\underline{\nabla }\cdot \left(\rho \underline{V}\underline{V}\right)+\underline{\nabla }p=\underline{\nabla }\cdot \left[2\mu {\left(\underline{\nabla }\underline{V}\right)}_{o}^{s}\right]+\rho \underline{a}+{\underline{S}}_{m},$$ (3)
• enthalpy
  $$\begin{array}{c} \rho \frac{Dh}{Dt}-\frac{Dp}{Dt}=\underline{\nabla }\cdot (\lambda \underline{\nabla }T)+\underline{\nabla }\cdot \left(\rho \sum_{i}h_{i}D_{i}\underline{\nabla }Y_{i}\right)+2\mu {\left(\underline{\nabla }\underline{V}\right)}_{o}^{s}:{\left(\underline{\nabla }\underline{V}\right)}_{o}^{s}+\rho \underline{a}\cdot \underline{V}+S_{e}, \end{array}$$ (4)

where

$${\left(\underline{\nabla }\underline{V}\right)}_{o}^{s}={\left(\underline{\nabla }\underline{V}\right)}^{s}-\frac{1}{3}(\underline{\nabla }\cdot \underline{V})\underline{\underline{I}}\;,\;{\left(\underline{\nabla }\underline{V}\right)}^{s}=\frac{\underline{\nabla }\underline{V}+\underline{\nabla }{\underline{V}}^{T}}{2},$$ (5)

and $\rho ,\;p,\;\underline{V},\;T$, and *h* are the mixture (fluid) density, pressure, velocity, temperature, and enthalpy, respectively. The vector quantity $\underline{a}$ accounts for the acceleration involved in the present problem: the component along z being the classical gravity $g=-9.81\;\;{m/s}^{2}$, the other two components along x and y, representing a variation of velocity in the direction of the train motion and a centrifugal contribution in a direction perpendicular to it (due to the curvature of the train trajectory), respectively. The symbol *Y_i_* indicates the non-dimensional mass fraction of the different components (the subscript v stands for vapor). The terms $S_{v},\;S_{m}$, and *S_e_* account for the exchange of mass, momentum, and energy between the carrier fluid and dispersed droplets, respectively, which implicitly indicates that in the present work the coupling between the gas and the dispersed liquid phase is of a two-way coupling nature (as we will illustrate in detail in Sec. II B where precise expressions for these terms are provided).

Moreover, the density of the different species (only air and pure vapor here) is expressed as $\rho_{i}=\rho Y_{i}$; the symbol *D_i_* denotes the species diffusion coefficient.

Accordingly, the following relationships also apply to the present problem:

$$\rho =\sum_{i}\rho_{i}\to \sum_{i}Y_{i}=1,$$ (6)

$$\begin{array}{c} h=\sum_{i}h_{i}Y_{i}=\sum_{i}\left(C_{pi}T\right)Y_{i}=C_{p}T\;,\;\\ \mu =\sum_{i}\mu_{i}Y_{i}\;,\;\lambda =\sum_{i}\lambda_{i}Y_{i}\;,\;C_{p}=\sum_{i}C_{pi}Y_{i}, \end{array}$$ (7)

where *μ*, *λ*, and *C_p_* are the mixture dynamic viscosity, thermal conductivity, and specific heat at constant pressure, respectively. Furthermore, the gas state equation can be cast in compact form as

$$p=\rho RT,\;\text{where}\;R=\sum_{i}R_{i}Y_{i},$$ (8)

or in an equivalent way as

$$p=\sum_{i}R_{i}\rho Y_{i}T=\sum_{i}\rho_{i}R_{i}T=\sum_{i}p_{i},$$ (9)

generally known as Dalton's law of partial pressures, where *R_i_* and *p_i_* represent the gas constant and the partial pressure of each component.

#### 1. Low-Mach-number asymptotics

For the considered problem (relatively small value of the Mach number, i.e., *M *=* *0.06), these equations are too complex and broad in scope. In particular, a well-known issue relating to their numerical integration is the need to keep the time integration step sufficiently small in order to capture properly the so-called acoustic wave propagation scale (a failure in doing so typically resulting in algorithm instability).

A convenient approach to circumvent this bottleneck (on which much commercial software rely, including the STAR-CCM+ v. 2021.1 platform at the root of the present study) is based on the so-called pressure splitting approach and the resulting simplifications that can be implemented in Eqs. (1)–(9) after filtering out the acoustic waves and replacing all the problem variables with equivalent series expansions in terms of the Mach number.

A rigorous justification for this modus operandi can be rooted in the study by Roller and Munz,^42^ where by means of multiple space scale asymptotic analysis it was shown that, in general, “the pressure” of a fluid can be decomposed into three parts with different physical meanings, these accounting separately for thermodynamic effects, acoustic wave propagation, and the balance of forces (dynamic pressure).

In such a context, Müller^43^ could show that acoustics removal from the equations directly leads (with proper mathematical developments) to the so-called low-Mach-number equations, which allow for large temperature and density changes as opposed to the standard Boussinesq equations. Such mathematical developments consist of expanding in power series law of the small parameter $M^{2}\ll 1$ (where, obviously, *M* is the reference Mach number) all the primitive variables as follows:

$$\rho =\rho_{0}^{*}+\rho_{1}^{*}M^{2}+O\left[{\left(M^{2}\right)}^{2}\right],$$ (10a)

$$p=p_{0}^{*}+p_{1}^{*}M^{2}+O\left[{\left(M^{2}\right)}^{2}\right],$$ (10b)

$$\underline{V}={\underline{V}}_{0}^{*}+{\underline{V}}_{1}^{*}M^{2}+O\left[{\left(M^{2}\right)}^{2}\right],$$ (10c)

$$T=T_{0}^{*}+T_{1}^{*}M^{2}+O\left[{\left(M^{2}\right)}^{2}\right].$$ (10d)

The reader specifically interested in this procedure may consider Beccantini *et al.*^44^ and Benteboula and Lauriat.^45^ Here, we limit ourselves to mentioning that after substituting the above expansions in the fully compressible Navier–Stokes equations, the lowest order terms in *M*^2^ are collected. At the order −1, the momentum equation reduces to

$$\underline{\nabla }p_{0}^{*}=0.$$ (11)

Moreover, at the order zero, the following low-Mach-number governing equations are obtained for mass and momentum:

$$\underline{\nabla }\cdot \left(\rho_{0}^{*}{\underline{V}}_{0}^{*}\right)=-\frac{\partial \rho_{0}^{*}}{\partial t^{*}},$$ (12)

$$\begin{array}{c} \frac{\partial }{\partial t^{*}}\rho_{0}^{*}{\underline{V}}_{0}^{*}+\underline{\nabla }\cdot \left(\rho_{0}^{*}{\underline{V}}_{0}^{*}{\underline{V}}_{0}^{*}\right)+\underline{\nabla }p_{1}^{*}=\underline{\nabla }\cdot \left[2\mu_{0}^{*}{\left(\underline{\nabla }{\underline{V}}_{0}^{*}\right)}_{o}^{s}\right]+\\ \rho_{0}^{*}\underline{a}+S_{m}, \end{array}$$ (13)

with the state equation

$$p_{0}^{*}=\rho_{0}^{*}RT_{0}^{*},$$ (14)

being required to determine the density $\rho_{0}^{*}(r,t)$.

These simplified equations are particularly useful as they make immediately clear that in the framework of the low-Mach-number approximation, pressure can be articulated into two components only: a thermodynamic pressure *p*_0_ homogenous in space and allowed to vary in time, and a dynamic pressure *p*_1_ decoupled from density and temperature fluctuations. The most remarkable implication of such a decomposition is that the dynamic pressure can be determined numerically using an approach similar to that traditionally implemented for incompressible flows in the framework of pressure-based methods; that is, the computation of the velocity can be split into three main steps. In the following, in order to illustrate this approach, we omit the asterisk and the subscripts related to the asymptotic expansions, with the zero- and first-order pressure contributions *p*_0_ and *p*_1_ being simply indicated for clarity as *P* and *p′*, respectively.

In an initial stage, an intermediate momentum field is computed solving an incomplete version of the momentum equation (deprived of the gradient of dynamic pressure)

$$\frac{\partial }{\partial t}(\rho {\underline{V}}_{\mathrm{int}}+\underline{\nabla }\cdot \left(\rho {\underline{V}}_{\mathrm{int}}{\underline{V}}_{\mathrm{int}}\right)=\underline{\nabla }\cdot \left[2\mu {\left(\underline{\nabla }{\underline{V}}_{\mathrm{int}}\right)}_{o}^{s}\right]+\rho \underline{a}+S_{m}.$$ (15)

In a second stage, the intermediate momentum field is formally corrected as

$$\rho^{n+1}{\underline{V}}^{n+1}={\left(\rho \underline{V}\right)}_{\mathrm{int}}-\Delta t\underline{\nabla }p\prime .$$ (16)

Where the dynamic pressure comes from the solution of an elliptic equation for pressure obtained by forcing Eq. (16) in Eq. (12),

$$\Delta t\nabla^{2}p\prime =\underline{\nabla }\cdot {\left(\rho \underline{V}\right)}_{\mathrm{int}}+\frac{\partial \rho }{\partial t}.$$ (17)

The time derivative of the density appearing in this equation, in turn, is computed separately determining the density at different instants through the gas state equation (where the thermodynamic pressure *P* appears), that is,

ρ=P/RT. (18)

Problem closure finally requires that the thermodynamic (constant in space) pressure is determined through a global balance of mass across the entire computational domain, that is,

$$P/R\int_{t=0}^{t}\frac{d\Omega }{T}=\rho_{t=0}\Omega +\int_{t=0}^{t}\left({\dot{m}}_{in}-{\dot{m}}_{\mathrm{out}}\right)dt,$$ (19a)

for a finite-size domain (where the dotted m denotes the incoming or outgoing mass flow rate) or

$$P=p_{\mathrm{atm}},$$ (19b)

for an unbounded domain.

As correctly reported by Munz *et al.*,^46^ in general, these methods are relatively robust.

#### 2. Turbulence model

In addition to the strategies described in Sec. II A 1, a specific model is also used in this work to account for turbulence effects typically associated with the examined value of the Reynolds number (in the range between 4600 and 16 500). Given the nature of the considered carrier flow (it being steady “in mean”), in particular, a method pertaining to the general class of RANS-based technique is used (where the acronym RANS stands for Reynolds Averaged Navier–Stokes Equations).

Such a category of methods generally relies on the idea that the velocity of the fluid at any point can be decomposed into two contributions: one obtained as a time-averaged value and another formally representing an instantaneous fluctuation with respect to the mean value, that is, $\underline{V}={\underline{V}}_{\mathrm{timeaveraged}}+\underline{V}\prime$, where obviously $\underline{V}\prime =\underline{V}-{\underline{V}}_{\mathrm{timeaveraged}}$ and ${\left(\underline{V}\prime \right)}_{\mathrm{timeaveraged}}={\underline{V}}_{\mathrm{timeaveraged}}-{\underline{V}}_{\mathrm{timeaveraged}}=0$.

Substituting these expressions into the original governing equations and taking a time (or ensemble) average yields the aforementioned (RANS) equations. A new term appears at the right-hand side of the momentum equation inside the divergence term, which formally plays the role of an additional stress in the flow. The extra term $-\underline{V}\prime \underline{V}\prime$ is known as “Reynolds stress tensor.”

Closure of the problem is then obtained by introducing the concept of turbulent viscosity by which the extra stress tensor is mathematically put in connection with the gradient of time-averaged velocity. The turbulent viscosity, in turn, is expressed as a function of the turbulent kinetic energy and the dissipation, which require two additional balance equations (to be solved together with the balance equations for the considered species, mass, momentum, and energy). In addition to the Reynolds stress tensor, other modifications are also implemented accordingly in the species and energy equations through the concepts of turbulent Prandtl and Schmidt numbers. Indeed, also the temperature and the vapor concentration can be split into a time-averaged contribution and a fluctuating part. Substitution of them into the original energy and species equations yields an additional term for each equation, which can formally be expressed as the product of a turbulent transport property and the gradient of the time-averaged version of the considered variable. In turn, these turbulent transport properties can be determined as a function of the turbulent kinematic viscosity through the concepts of turbulent Prandtl and Schmidt numbers (both assumed to be equal to 0.9 in the present work). In particular, here the Realizable k−ε turbulence model (Shih *et al.*^47^) has been adopted.

### B. Lagrangian discrete phase

As anticipated in the introduction, we treat saliva droplets as Lagrangian particles. As the considered number of droplets is relatively limited and their size is small, interactions between the particles (collisions/agglomerations) are neglected; the presence of other droplets, or solid particles, that may be dispersed in the air is also neglected. Accordingly, each droplet is tracked individually throughout the computational domain. Although this approach may look less convenient than other methods where all coexisting phases are dealt with in the framework of a single Eulerian treatment (typically based on the introduction of a volume of fraction variable or similar concepts, see, e.g., Capobianchi *et al.*^48^ and Lappa^49^), the hybrid Eulerian–Lagrangian has distinct advantages, which make it particularly suitable (see, e.g., Capobianchi and Lappa;^50^ Lappa and Burel;^51^ and Lappa^52^) for the analysis of the problems like that being addressed in the present work. For each droplet, in particular, a set of 3 differential equations are solved, which describe the evolution of its position (and velocity), mass, and temperature, respectively. The additional details on the different terms appearing in these equations are provided in an ordered fashion in Subsections II B 1–II B 6.

#### 1. Particle equation of motion

When the conservation equation of momentum for a particle is written in the Lagrangian framework, the change in momentum is balanced by surface and body forces that act on the particle as expressed by the right-hand side of Eq. (20) (where n is the number of forces and k is an index used to indicate the generic force)

$$m_{p}\frac{d{\underline{V}}_{p}}{dt}=\sum_{k=1}^{n}{\underline{F}}_{k}.$$ (20)

#### 2. Drag force

The drag force can be expressed as follows:

$${\underline{F}}_{d}=\frac{1}{2}C_{d}\rho A_{p}|{\underline{V}}_{s}|{\underline{V}}_{s},$$ (21)

where ${\underline{V}}_{s}=\underline{V}-{\underline{V}}_{p}$ is the particle slip velocity and the drag coefficient *C_d_* is a function of the small-scale flow features around the individual particles, which can be derived with the *Schiller–Naumann correlation* (Schiller and Naumann^53^).

#### 3. Pressure gradient force

The pressure gradient force reads

$${\underline{F}}_{p}=-v_{p}\underline{\nabla }p_{\mathrm{static}},$$ (22)

where *v_p_* is the volume of the particle and $\underline{\nabla }p_{\mathrm{static}}$ is the gradient of the static pressure in the continuous phase.

#### 4. Virtual mass

The expression for the virtual mass force can be cast in compact form as follows:

$${\underline{F}}_{vm}=C_{vm}\rho v_{p}\left(\frac{D\underline{V}}{Dt}-\frac{d{\underline{V}}_{p}}{dt}\right),$$ (23)

where *C_vm_* is the virtual mass coefficient and the operator *D*/*Dt* denotes the substantial derivative.

#### 5. Turbulent dispersion

At this stage, it should also be pointed out that, in addition to the standard forces described before, a particle in turbulent flow can also experience what is generally known as turbulent dispersion, or simply “particle dispersion.” This phenomenon is a natural consequence of turbulence itself and the extra forces that it creates at the microscopic level as a result of turbulent momentum exchange. These tend to spread discrete solid particles or droplets exhibiting inertia and a mean relative fluid–particle velocity due to gravity. Lagrangian stochastic (LS) models traditionally used to account for this process can be split into two main categories, namely, the eddy interaction model (EIM) and the random flight or walk model (RWM). They can be distinguished essentially according to the strategy implemented to statistically generate the turbulent fluid velocity in the particle surrounding [this being necessary to solve Eq. (21), i.e., the Lagrangian equation of particle motion, Huilier^54^].

In particular, here we follow Gosman and Ioannides,^55^ where this is achieved assuming that any particle passes through a sequence of turbulent eddies. The underlying concept is that the interactions between a particle and a succession of fluid eddies can be characterized by three parameters, namely, an eddy instantaneous velocity, an eddy lifetime, and an eddy size (these being functions of the considered flow Reynolds number). A Monte Carlo (MC) process is typically associated with this process. The turbulent velocity is sampled randomly from a Gaussian probability distribution function with a standard deviation, and it is kept constant for a given time of the order of the eddy lifetime. The reader interested in a more complete description of this approach, and the related assumptions and the related logical sequence of steps may consider the very recent review by Huilier.^54^

#### 6. Droplet mass and heat transfer

Following a common practice in the literature, in order to mimic properly saliva droplets emitted by human beings, dispersed droplets are assumed to be composed of a water-NaCl solution. We wish to remark that, obviously, in the framework of this approximation, mucus and other viscoelastic substances potentially present in the saliva of a sick person are not taken into account. Although these substances may have an impact on the evaporation process, unfortunately reliable data on their percentages and related influence on evaporation are not available yet. The water-NaCl solution-based evaporation model traditionally assumed for saliva droplets can be found in Chaudhuri *et al.*,^25^ and it will be briefly outlined in this subsection. In particular, here we limit ourselves to presenting the related governing equations where the subscripts 1, 2, and 3 denote water, air, and salt, respectively. The droplet water mass change due to evaporation can be expressed as

$$\begin{array}{c} \frac{\partial m_{1}}{\partial t}=-4\pi \rho_{v}D_{v}R_{s}\;\text{log}\;(1+B_{M})-4\pi \rho_{v}\alpha_{g}R_{s}\;\text{log}\;(1+B_{T}). \end{array}$$ (24)

*ρ_v_* is the density of the water vapor, *D_v_* is the binary diffusivity of water vapor in air, *R_s_* is the droplet radius, and *α_g_* is the thermal diffusivity of the air. $B_{M}=(Y_{1,s}-Y_{1,\infty })/(1-Y_{1,s})$ and $B_{T}=C_{p,l}(T_{s}-T_{\infty })/h_{fg}$ are the Spalding mass transfer and heat transfer numbers. *Y*_1_ is the mass fraction of water vapor, while subscripts *s* and ∞ indicate the location (droplet surface and far field). $C_{p,l}$ and *h_fg_* are the specific heat and specific latent heat of vaporization of the droplet liquid. Using the Raoult's law, the vapor pressure at the droplet surface for the binary solution can be determined as

$$P_{\mathrm{vap}}(T_{s},\chi_{1,s})=\chi_{1,s}P_{\mathrm{sat}}(T_{s}),$$ (25)

where $\chi_{1,s}=1-\chi_{3,s}$ is the mole fraction at the droplet surface in the liquid phase. Considering the effect of Raoult's law and relative humidity, the vapor concentration at the droplet surface and at the far field can therefore be obtained as

$$Y_{1,s}=\frac{P_{\mathrm{vap}}(T_{s},\chi_{1,s})M_{1}}{P_{\mathrm{vap}}(T_{s},\chi_{1,s})M_{1}+(1-P_{\mathrm{vap}}(T_{s},\chi_{1,s}))M_{2}},$$ (26)

$$Y_{1,\infty }=\frac{(RH)P_{\mathrm{sat}}(T_{\infty })M_{1}}{(RH)P_{\mathrm{sat}}(T_{\infty })M_{1}+(1-(RH)P_{\mathrm{sat}}(T_{\infty }))M_{2}},$$ (27)

where *M*_1_ and *M*_2_ are the molecular weights of water and air.

As for the droplet energy balance, it has been verified that the thermal gradient in the liquid phase is rather small for the conditions considered in the present work, and thus, it can be formulated as

$$m_{l}C_{p,l}\frac{\partial T_{s}}{\partial t}=-k_{g}A_{s}\frac{\left(T_{s}-T_{\infty }\right)}{R_{s}}+\frac{\partial m_{1}}{\partial t}h_{fg}-\frac{\partial m_{1}}{\partial t}e_{l},$$ (28)

where *T_s_* is the droplet temperature, $m_{l}=(4/3)\pi \rho_{l}R_{s}^{3}$ and $A_{s}=4\pi R_{s}^{2}$ are the droplet mass and surface area, *ρ_l_* and *e_l_* are the density and specific internal energy of the mixture of salt and water, and *k_g_* is the conductivity of the air, respectively.

Yet, in line with the existing literature and very recent efforts on this subject, we also take into account that if the concentration of solute inside the droplet exceeds a given threshold (due to water evaporation), crystallization phenomena are enabled (Ranz and Marshall^56^). Considering that $P_{\mathrm{vap}}(T_{s},\chi_{1,s})$ is also a function of the salt concentration in the droplet, these phenomena can be modeled through a dedicated (droplet-related) species balance equation, that is,

$$\frac{dmY_{3}}{dt}+{\dot{m}}_{3,\mathrm{out}}=0,$$ (29)

where *Y*_3_ is the dissolved salt mass fraction and ${\dot{m}}_{3,\mathrm{out}}$ is the solute mass that leaves the solution due to crystallization. When the supersaturation ratio $S=Y_{3}/Y_{3,c}$, with $Y_{3,c}=0.393$ (Gregson *et al.*^57^), is greater than 1, crystallization begins. In the present work, the growth rate of the crystal is modeled using the simplified rate equation (Naillon *et al.*^58^ and Derluyn *et al.*^59^)

$$\frac{dl}{dt}={\left(S-1\right)}^{g_{cr}}C_{cr}e^{-E_{a}/RT_{s}},$$ (30)

where *l* is the crystal radius, $C_{cr}=1.14\times 10^{4}\;m/s,\;\;E_{a}=58\;180\;\;J/\text{mol}$, and *g_cr_* = 1. The rate of change of the crystal mass can therefore be finally determined as^58^

$${\dot{m}}_{3,\mathrm{out}}=\frac{dm_{3,\mathrm{crystal}}}{dt}=6\rho_{s}{\left(2l\right)}^{2}\frac{dl}{dt}.$$ (31)

#### 7. Coupling terms for the Eulerian equations

At this stage, we are in a condition to define precisely the coupling terms appearing in Eqs. (2)–(4) (by which the dispersed droplets can exert a back influence on the carrier flow). These read

$$S_{v}=-\sum_{k=1}^{n}\frac{1}{\delta \Omega }\frac{dm_{l,k}}{dt},$$ (32a)

$${\underline{S}}_{m}=-\sum_{k=1}^{n}\frac{m_{l,k}}{\delta \Omega }\frac{d{\underline{V}}_{p,k}}{dt}+\sum_{k=1}^{n}\frac{{\underline{V}}_{p,k}}{\delta \Omega }\frac{dm_{l,k}}{dt},$$ (32b)

$$S_{e}=-\sum_{k=1}^{n}\frac{m_{l,k}}{\delta \Omega }C_{p,l}\frac{dT_{s,k}}{dt},$$ (32c)

where δΩ denotes the volume of the generic control volume, n is the number of droplets contained in that volume, and k is an index used to indicate the generic particle. The sign minus in front of each summation obviously follows from the nature of the interphase exchange itself (an increase in the vapor concentration, carrier fluid momentum, or enthalpy must obviously correspond to a decrease in the droplet liquid mass, a decrease in the droplet momentum, and a shrinkage of the droplet temperature, respectively, and vice versa).

## III. VALIDATION

The validation process has been articulated in three stages. As a first step of this hierarchy, the case of a static evaporating droplet has been considered. Then, dynamic conditions (moving droplets) have been examined for both laminar and turbulent flows.

### A. Static validation

**FIG. 1.**
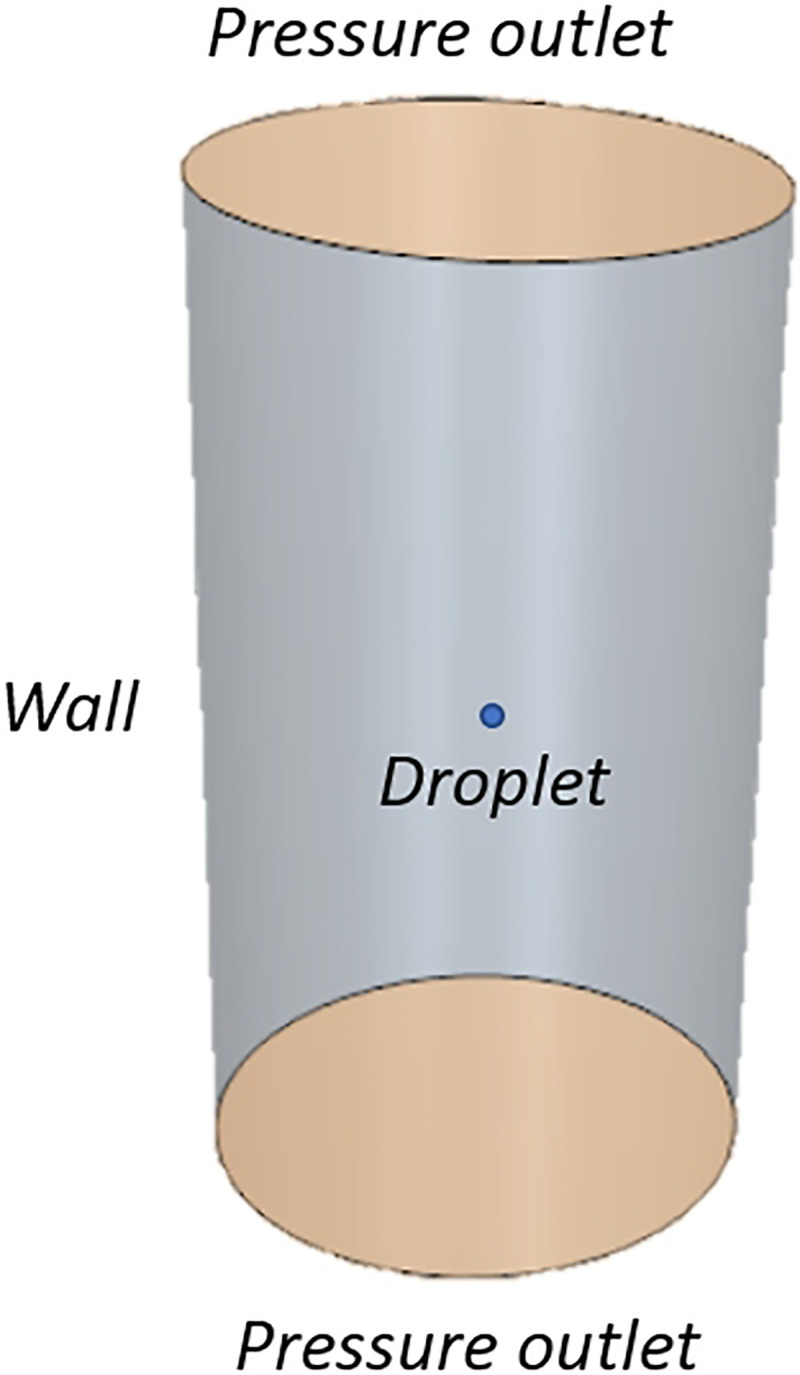
Numerical domain considered for static validation.

The evaporation and crystallization models illustrated in Sec. II have been implemented in the solver Star-CCM+ (see Polizio^60^) and have been correlated with the experimental data by Chaudhuri *et al.*^25^ The experimental setup involved a single particle suspended on an ultrasonic levitator (tec5), for which the diameter change was recorded. To mimic this case, in the numerical simulation, a cylindrical domain of 75 mm base diameter and 120 mm height has been generated, see Fig. 1. The boundary conditions are adiabatic wall on the side and pressure outlet at ambient pressure on the top and bottom faces. A polyhedral mesh of 3300 cells has been used to discretize this domain. The following environmental conditions have been assumed:

• Air temperature, 30 °C
• Relative humidity, 50%
• Air pressure, 1 atm
• No gravity effect

**FIG. 2.**
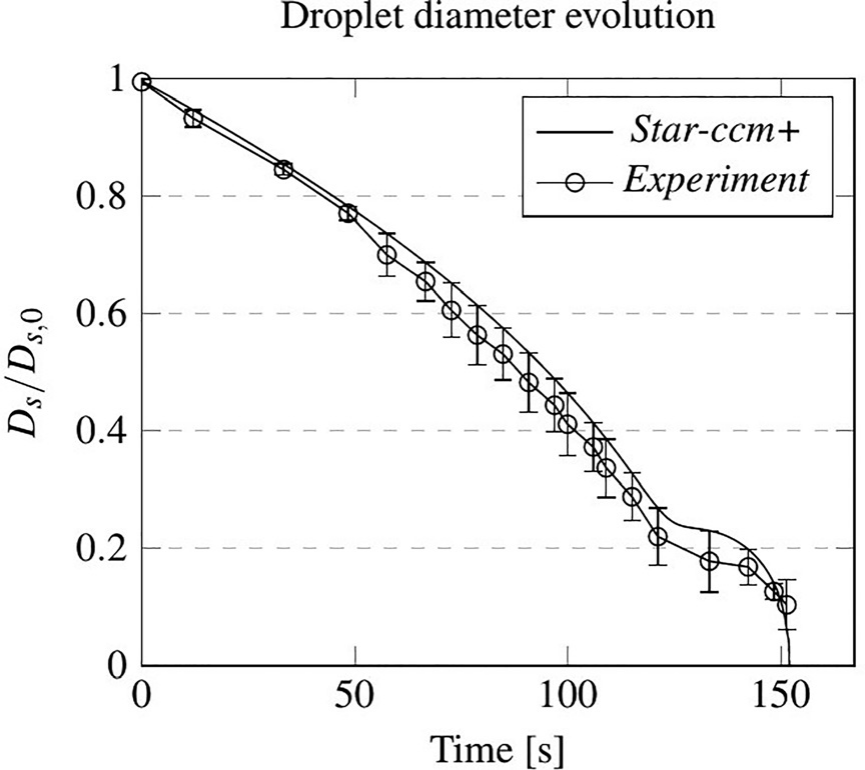
Static cross-validation: evolution of droplet diameter in time according to experimental data (with error bar) and present numerical simulation (solid line).

In order to reproduce the experiments, a single droplet has been inserted in the middle of the numerical domain, with no initial velocity. The droplet initial diameter is 338 μm and is composed of a water/salt solution (1% w/w). The evolution of the ratio between droplet diameter and droplet initial diameter is shown in Fig. 2.

As the reader will easily realize by inspecting this figure, the numerical results display good agreement with the experimental data. In particular, at about 130 s the equilibrium condition between droplet and air is reached, and evaporation becomes relatively weak or almost negligible. Then, crystallization occurs and the droplet diameter decreases again.

### B. Cross-validation

For the dynamic conditions (moving droplets), a slightly different validation strategy has been implemented. The results obtained with two different types of commercial CFD software *Fluent* and *Star-ccm+* have been compared for a fixed (relevant) reference case.

**FIG. 3.**
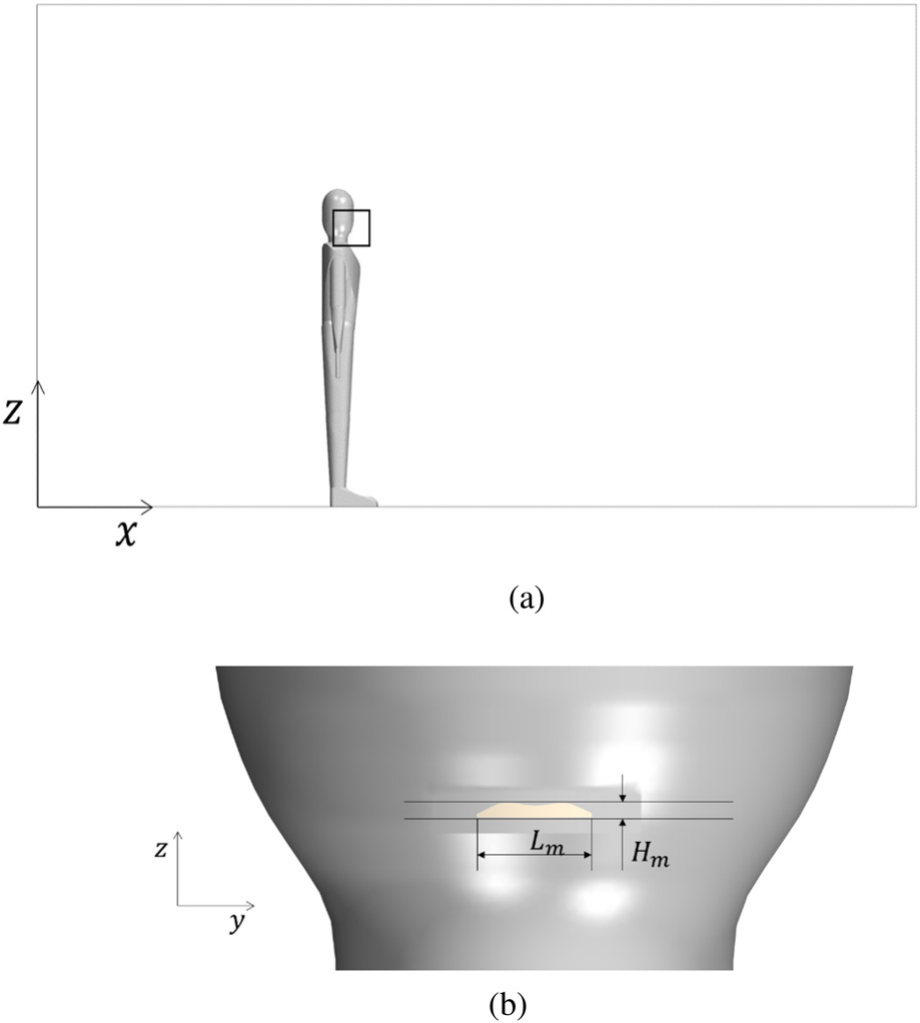
Geometrical model for cross-validation. (a) 2D sketch of the mannequin and the related computational domain (the mouth area highlighted using a black rectangle, is shown in detail in panel (b): length 6 m, width 2 m, height 3 m. (b) Shape of the mouth at *z *=* *1.64 m: *L_m_* = 4 cm, $L_{m}/H_{m}=8.26$.

In particular, the geometry shown in Fig. 3(a) has been implemented, discretized with a cell base size of 40 mm, locally refined around the mouth and torso areas, and simulated with the aforementioned commercial platforms (both relying on a time implicit formulation; moreover, for both cases, the convective terms have been discretized using a second-order upwind scheme). Further details are reported in the following.

As evident in the figure, the computational domain of length 6 m, width 2 m, and height 3 m includes a mannequin on the symmetry plane *y *=* *0 at the abscissa x=2 m. Following Dbouk and Drikakis,^5^ the mannequin average mouth print has been assumed to have a rectangular shape with an aspect ratio of $L_{m}/H_{m}=8.26$ and length *L_m_* = 4 cm [see Fig. 3(b)].

The entire sidewall is treated as a symmetry plane, while the top of the room is a pressure outlet boundary. The initial temperature of the room is T=20  °C, there is no air motion, and relative humidity is assumed to be initially zero. Droplet material is pure water.

At the initial time, a total number of 4000 particles are assumed to enter the domain through the mouth boundary shown in Fig. 3(b) with a total mass of 1.07 *μ*g. The particle initial condition can be gathered from Table I.

**TABLE I.** Initial condition for discrete phase: droplet material assumed as pure water, total injected mass is 1.07 *μ*g.

Number	4000
Diameter	80 *μ*m
Temperature	34 °C
Velocity	8 m/s

Xie *et al.*^61^ conducted experimental measurements and quantified exhaled droplet's mass and size due to talking and coughing. As reported by Dbouk and Drikakis,^5^ the Rosin–Rammler distribution law, also known as Weibull distribution, with mean diameter 80 *μ*m can be used to reproduce satisfactorily experimental measurements. In particular, here we consider an initial droplet diameter of 80 *μ*m.

#### 1. Laminar case

In the present subsection, the results concerning the numerical analysis of the laminar case are given in terms of the following properties of the Lagrangian phase:

• mass evaporation;
• evolution of center of gravity (CoG) coordinates.

**FIG. 4.**
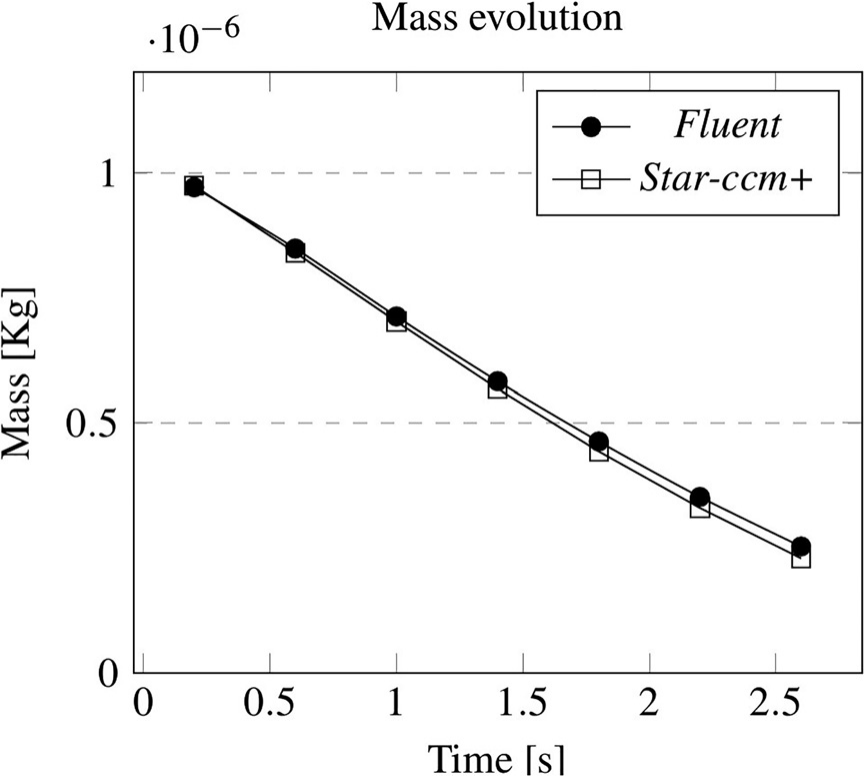
Cross-validation for the laminar case: evolution of mass in time.

**FIG. 5.**
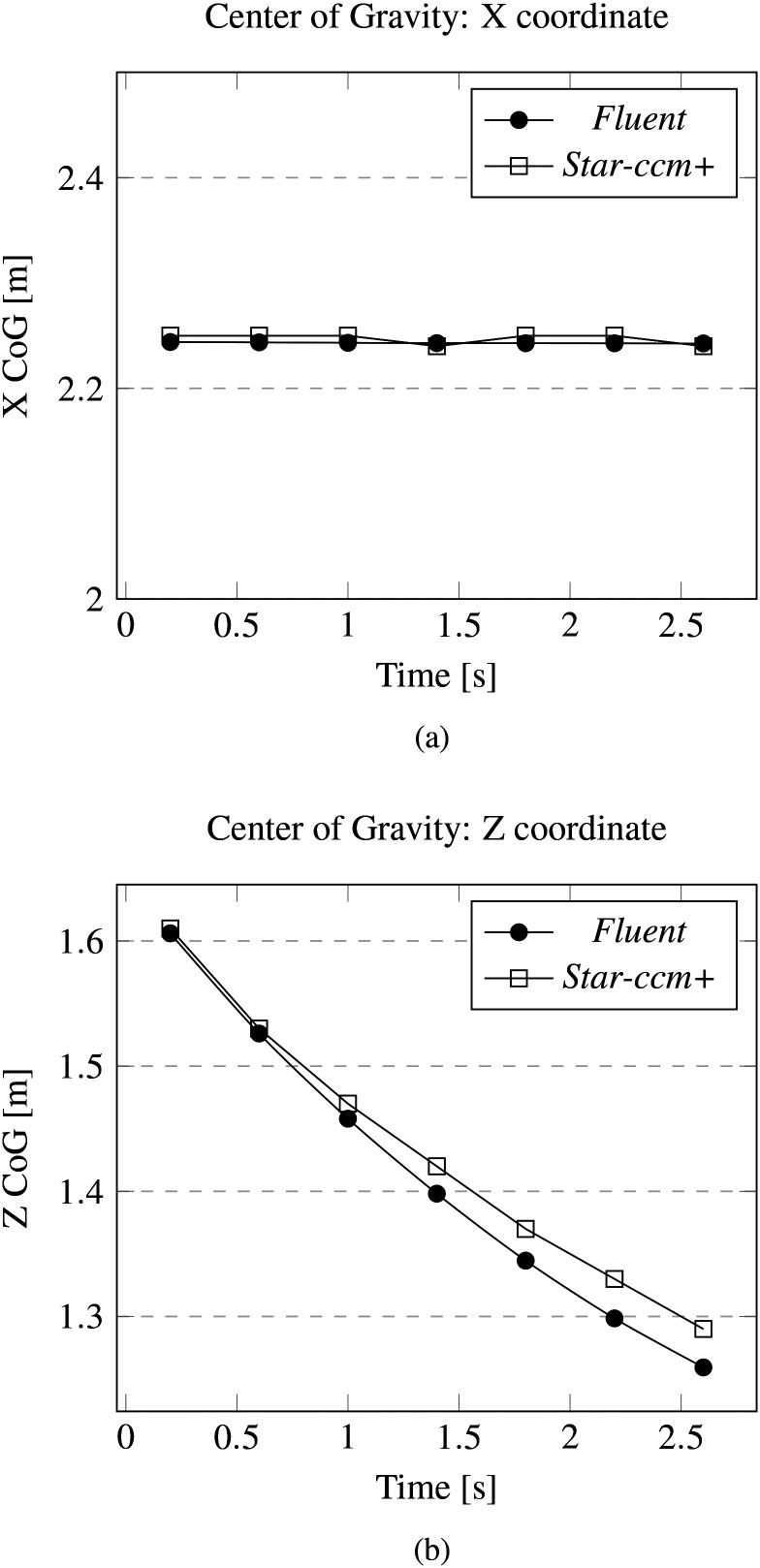
Cross-validation for the laminar case: evolution of the center of gravity (CoG) *x* and *z* coordinates. CoG *y* coordinates evolve on the symmetry plane. (a) CoG x coordinate. (b) CoG z coordinate.

Tables II and III summarize the numerical data and the relative percentage errors related to mass and kinematic parameters obtained performing the same droplet simulation with the two aforementioned commercial software. As also quantitatively substantiated by Figs. 4 and 5, the agreement is excellent.

**TABLE II.** Numerical results for the laminar case. Comparison of mass and relative percentage error obtained with *Fluent* and *Star-ccm*.

Time	Mass (mg)		Error (%)
	*Fluent*	*Star-ccm*	
0.2	0.97	0.97	0
0.6	0.85	0.84	1
1.0	0.71	0.70	1
1.4	0.58	0.57	3
1.8	0.46	0.44	4
2.2	0.35	0.33	7
2.6	0.25	0.23	11

**TABLE III.** Numerical results for the laminar case. Comparison of center of gravity coordinates and relative percentage error obtained with *Fluent* and *Star-ccm*.

Time	*X_CoG_* (m)		Error (%)	*Z_CoG_* (m)		Error (%)
	*Fluent*	*Star-ccm*		*Fluent*	*Star-ccm*	
0.2	2.24	2.25	0	1.61	1.61	0
0.6	2.24	2.25	0	1.53	1.53	1
1.0	2.24	2.25	0	1.46	1.47	1
1.4	2.24	2.24	0	1.40	1.42	1
1.8	2.24	2.25	0	1.34	1.37	2
2.2	2.24	2.25	0	1.30	1.33	2
2.6	2.24	2.24	0	1.26	1.29	2

**FIG. 6.**
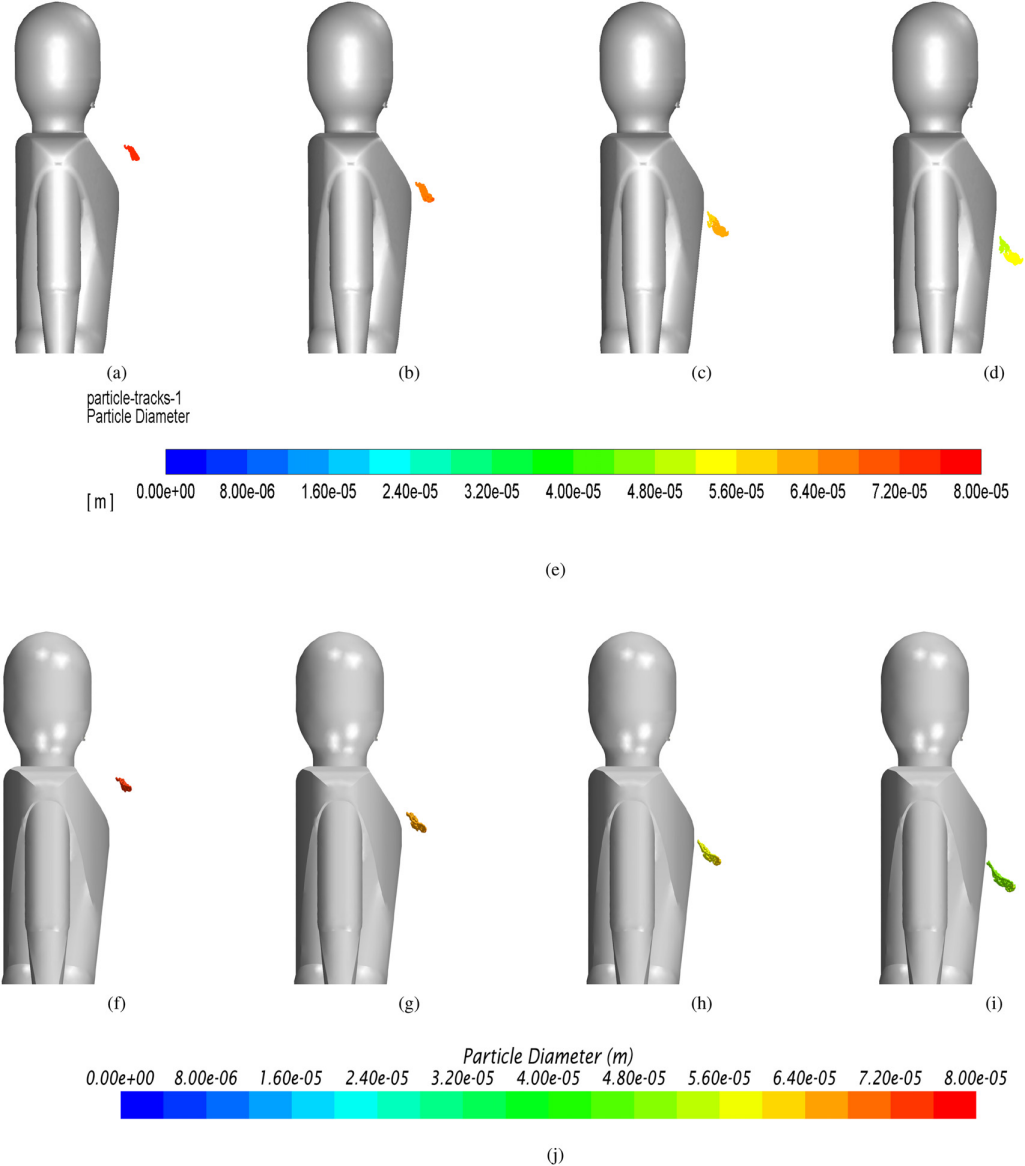
Laminar case: evolution of particle diameter at different solution times. (a) *Fluent* t = 0.6 s. (b) *Fluent* t = 1.2 s. (c) *Fluent* t = 1.8 s. (d) *Fluent* t = 2.4 s. (e) Contour legend *Fluent*. (f) *Star-ccm* t = 0.6 s. (g) *Star-ccm* t = 1.2 s. (h) *Star-ccm* t = 1.8 s. (i) *Star-ccm* t = 2.4 s. (j) Contour legend *Star-ccm*.

The agreement also holds in terms of the shape of the particle cloud, see Fig. 6.

#### 2. Turbulent case

**FIG. 7.**
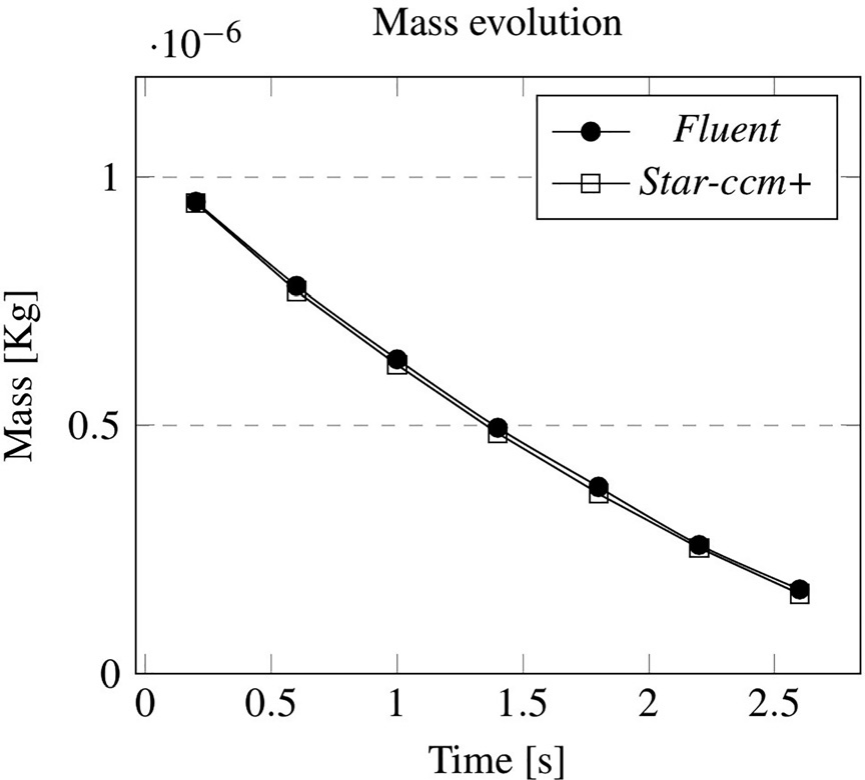
Cross-validation for the turbulent case: evolution of mass in time.

**FIG. 8.**
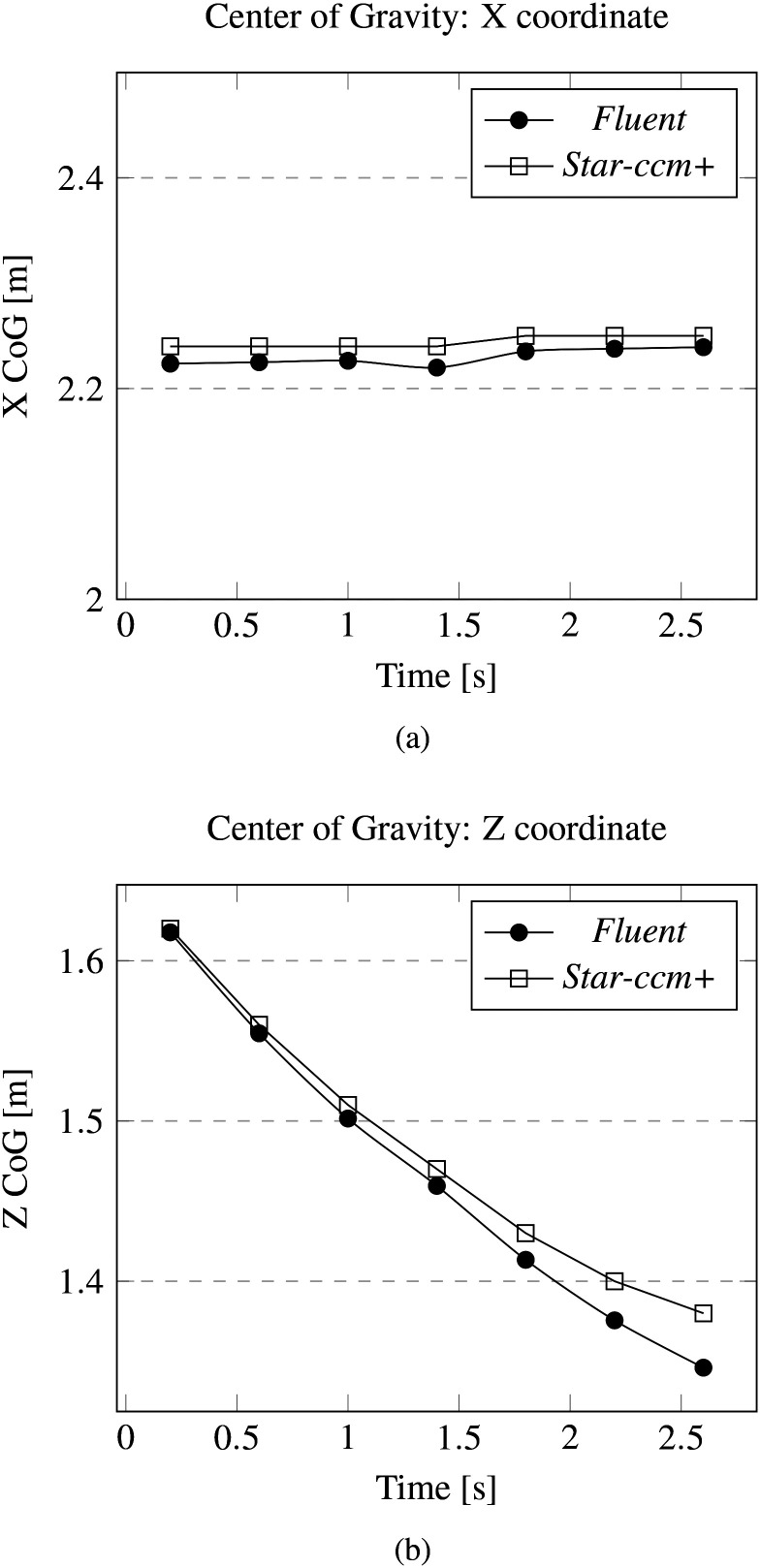
Cross-validation for the turbulent case: evolution of center of gravity (CoG) *x* and *z* coordinates. CoG *y* coordinates evolve on the symmetry plane. (a) CoG x coordinate. (b) CoG z coordinate.

**FIG. 9.**
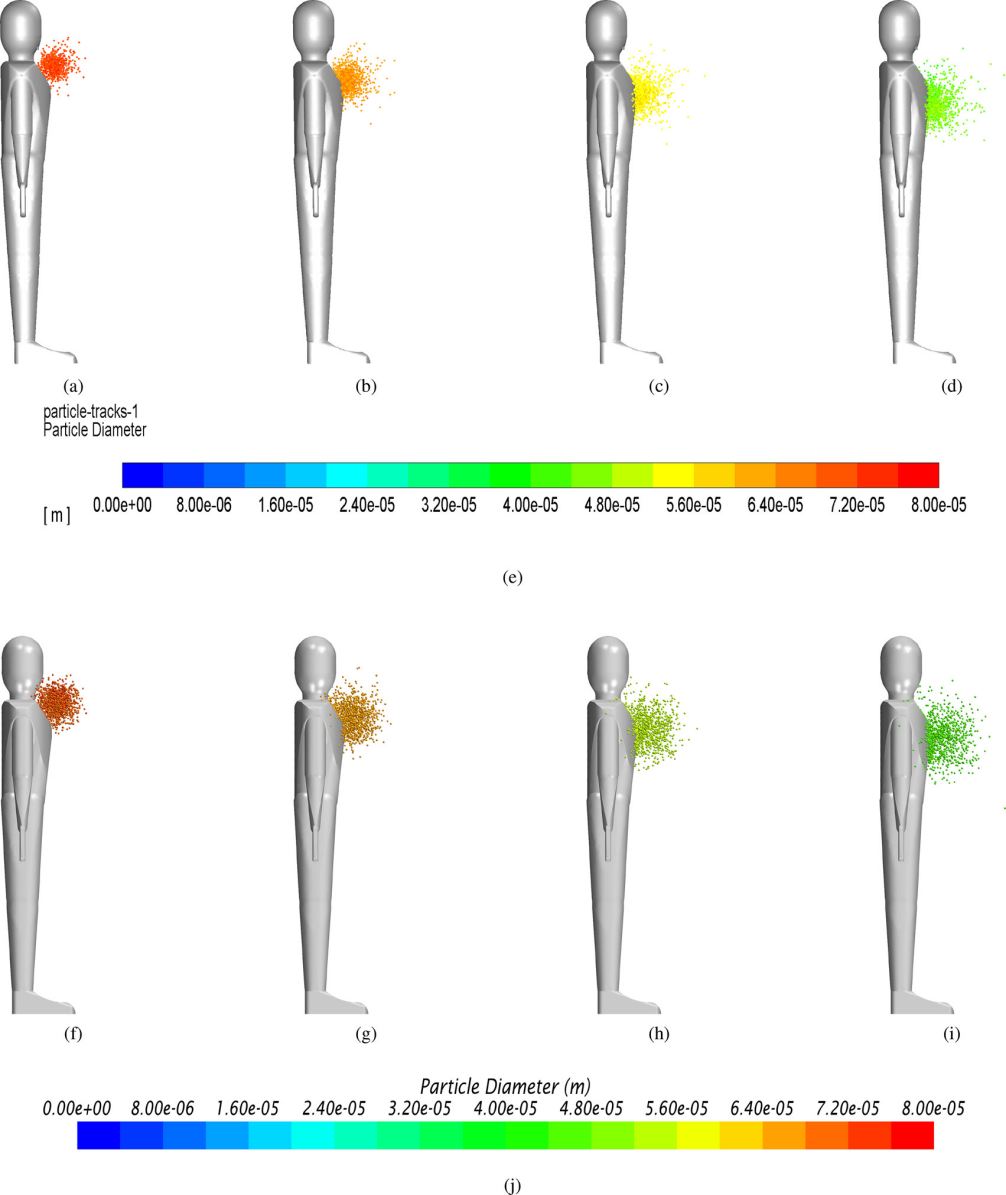
Turbulent case: evolution of particle diameter at different solution times. (a) *Fluent* t = 0.6 s. (b) *Fluent* t = 1.2 s. (c) *Fluent* t = 1.8 s. (d) *Fluent* t = 2.4 s. (e) Contour legend *Fluent*. (f) *Star-ccm* t = 0.6 s. (g) *Star-ccm* t = 1.2 s. (h) *Star-ccm* t = 1.8 s. (i) *Star-ccm* t = 2.4 s. (j) Contour legend *Star-ccm*.

The adopted turbulence model is the standard k−ε for both computational platforms; moreover, turbulence dispersion acts on the discrete phase. Following the same approach undertaken for the laminar case, the numerical data and relative percentage errors have been collected in Tables IV and V. The trend of the monitored parameters is shown in Figs. 7 and 8. It can be seen that the agreement between the two platforms is excellent also in this case and holds even in terms of the shape of the particle cloud, see Fig. 9.

**TABLE IV.** Numerical results for the turbulent case. Comparison of mass and relative percentage error obtained with *Fluent* and *Star-ccm*.

Time	Mass (mg)		Error (%)
	*Fluent*	*Star-ccm*	
0.2	0.95	0.95	0
0.6	0.78	0.77	0
1.0	0.63	0.62	0
1.4	0.49	0.48	2
1.8	0.38	0.36	4
2.2	0.26	0.25	2
2.6	0.17	0.16	6

**TABLE V.** Numerical results for the turbulent case. Comparison of center of gravity coordinates and relative percentage error obtained with *Fluent* and *Star-ccm*.

Time	*X_CoG_* (m)		Error (%)	*Z_CoG_* (m)		Error (%)
	*Fluent*	*Star-ccm*		*Fluent*	*Star-ccm*	
0.2	2.22	2.24	1	1.62	1.62	0
0.6	2.22	2.24	1	1.55	1.56	0
1.0	2.23	2.24	1	1.50	1.51	1
1.4	2.22	2.24	1	1.46	1.47	1
1.8	2.24	2.25	1	1.41	1.43	1
2.2	2.24	2.25	1	1.37	1.40	2
2.6	2.24	2.25	1	1.34	1.38	2

## IV. TRAIN CABIN

As anticipated in the introduction, to fill a gap in the literature, in the present work we consider an interregional train passenger compartment. In order to make the outcomes of the numerical study “realistic” as much as possible, the various components and sub-systems of such a compartment are modeled in detail (as further illustrated in Subsections IV A–IV C).

### A. Heating, Ventilation, and Air Conditioning (HVAC) system

**FIG. 10.**
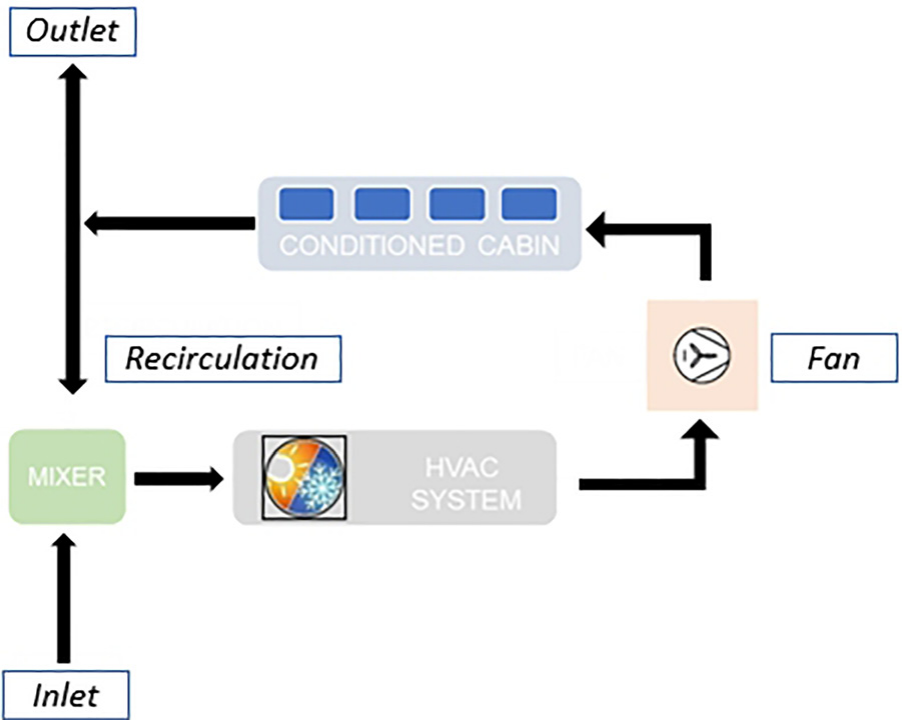
Schematic representation of the HVAC system working principle.

Air entering the passenger cabin must be conditioned to ensure a certain degree of thermal comfort for the occupants. In general, thermal comfort can be assessed in terms of existing EN standard requirements on air velocity and temperature distribution. For the case being, the applicable standard is the EN13129.^62^ The general working principle of an HVAC (heating, ventilation, and air conditioning) system is illustrated in Fig. 10. The key elements of an HVAC system are as follows:

• *INLET*: air mass flow rate of fresh air coming from external ambient toward a mixing area;
• *RECIRCULATION*: air mass flow rate coming from the conditioned cabin toward a mixing area;
• *OUTLET*: air mass flow coming from conditioned cabin driven out to the ambient and replaced by fresh air;
• *MIXER*: it combines recirculation and fresh air;
• *HVAC system*: provides the mixed air with the correct characteristics (temperature and relative humidity) before entering the passenger coach;
• *FAN*: is the component driving the treated air into the cabin;
• *CONDITIONED CABIN*: is the monitored thermal comfort area.

In order to define the main characteristics of an HVAC system, an evaluation of the thermal load in the conditioned cabin has to be performed. The thermal power is composed mainly of sensible and latent heat as follows:

• SENSIBLE HEAT (*Q_sens_*), thermal load due to temperature changes:
  – Conductive heat;
  – external power exchange;
  – human sensible power production;
  – lighting/system power;
  – thermal leakage;
  – refresh air.
• LATENT HEAT (*Q_lat_*), thermal load due to humidity changes:
  – people production;
  – relative humidity changes due to electrical components;
  – evaporation/condensing process;
  – fresh air.

**FIG. 11.**
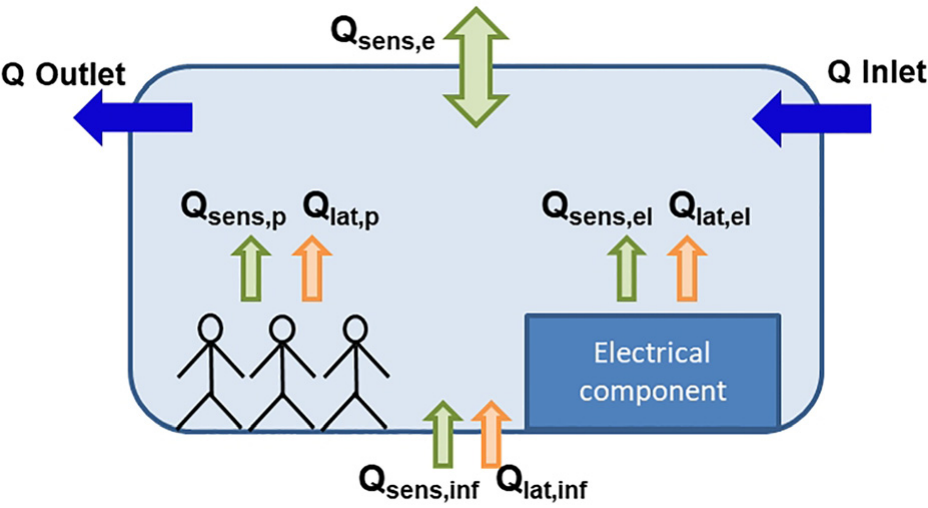
Schematic representation of cabin control volume for HVAC power evaluation.

In particular, the power required by the HVAC system can be evaluated by applying the heat balance equation to the cabin control volume sketched in Fig. 11; this reads

$$Q_{\mathrm{tot}}=Q_{\mathrm{sens},\mathrm{tot}}+Q_{\mathrm{lat},\mathrm{tot}},$$ (33)

$$Q_{\mathrm{sens},\mathrm{tot}}=Q_{\mathrm{sens},e}+Q_{\mathrm{sens},p}+Q_{\mathrm{sens},el}+Q_{\mathrm{sens},\inf},$$ (34)

$$Q_{\mathrm{lat},\mathrm{tot}}=Q_{\mathrm{lat},p}+Q_{\mathrm{lat},el}+Q_{\mathrm{lat},\inf},$$ (35)

where $Q_{\mathrm{sens},e}$ is the sensible heat exchanged with the external ambient, $Q_{\mathrm{sens}/\mathrm{lat},p}$ is the sensible/latent heat produced by human beings, $Q_{\mathrm{sens}/\mathrm{lat},el}$ is the sensible/latent heat produced by electrical components, and $Q_{\mathrm{sens}/\mathrm{lat},\inf}$ is the sensible/latent heat caused by air infiltration.

**FIG. 12.**
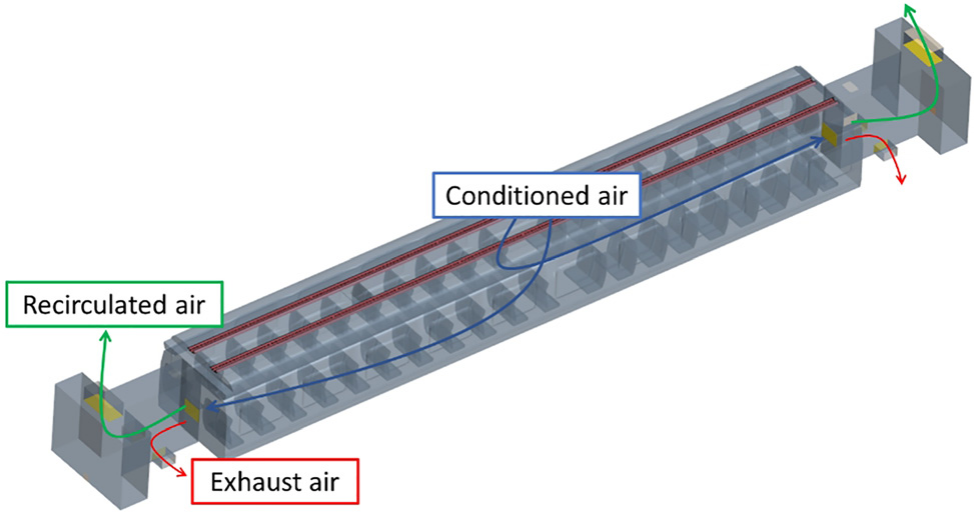
Schematic representation air path line.

For the particular case sketched in Fig. 12, the conditioned air enters the cabin, in which all comfort parameters have to be satisfied. Then, the air is forced to pass through the grid opening and enters the vestibules area; here, the volume flow rate is split in recirculated and exhaust streams. The recirculated air enters the recirculation ducts where it is mixed with fresh air and, after being treated again by the air conditioning system to meet comfort parameters, it enters the cabin comfort area, while exhaust air is released in the external environment.

### B. Standard requirement: EN13129

**FIG. 13.**
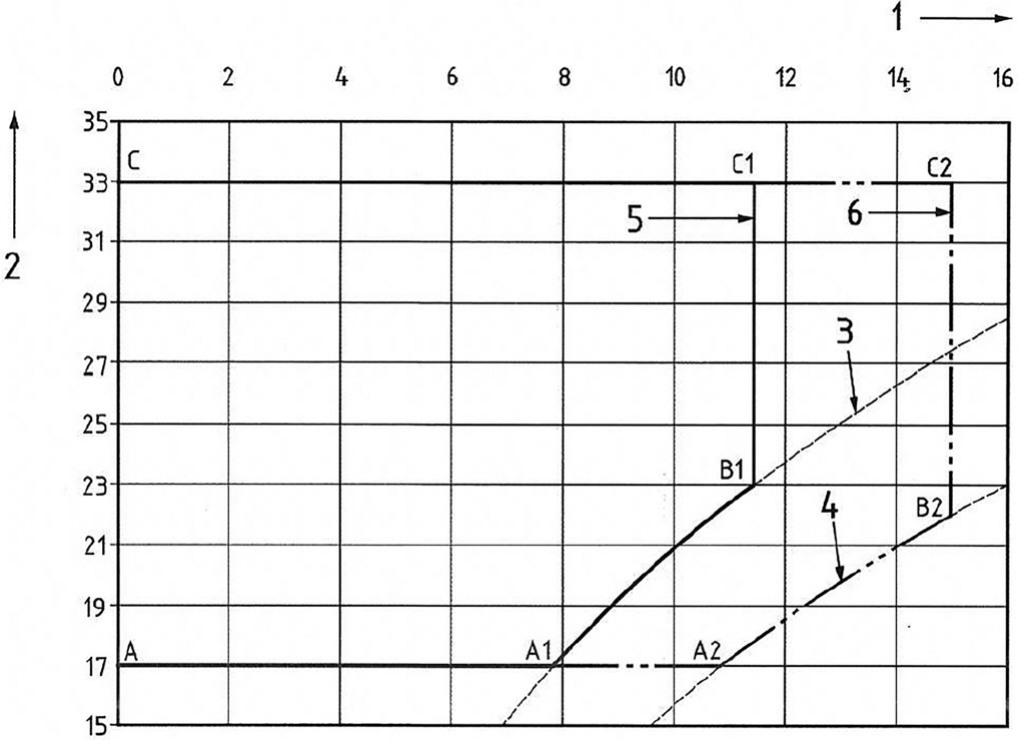
Comfort zone envelope as a function of interior air temperature and humidity as prescribed by EN13129.^62^ Legend: 1 absolute humidity (g/kg); 2 mean interior temperature (°C); 3 relative humidity line at 65%; 4 relative humidity line at 90%; 5 $1^{st}$-class quality range; 6 $2^{nd}$-class quality range.

The standard EN13129 imposes quality limits for air temperature and relative humidity. As shown in Fig. 13, air relative humidity must not exceed 65% and 90% for first class quality and second class quality limits, respectively. In general, both quality limits result in a decreasing acceptable level of air relative humidity with increasing air temperature.

Other important parameters relating to the HVAC system design imposed by the EN13129 standard are typically given in terms of external design conditions and internal temperature. Typical values for a temperate zone country are reported in Table VI.

**TABLE VI.** EN13129^62^ design condition requirements for a typical temperate climate country.

	*T_int_*^a^ (°C)	*T_ext_*^b^ (°C)
Summer	27	40
Winter	21	−20

^a^ Summer maximum internal temperature and minimum winter internal temperature.

^b^ External temperature design condition.

At this stage, we wish also to highlight that a “summer scenario” is not as critical as the winter one in terms of potential droplet propagation. Indeed, the droplet evaporation time is known to decrease drastically when the air temperature becomes higher. For this reason, only winter conditions are assessed in the present work as critical circumstances for droplet evolution (the reader being referred to the point B1 in Fig. 13).

### C. Computational domain

**FIG. 14.**
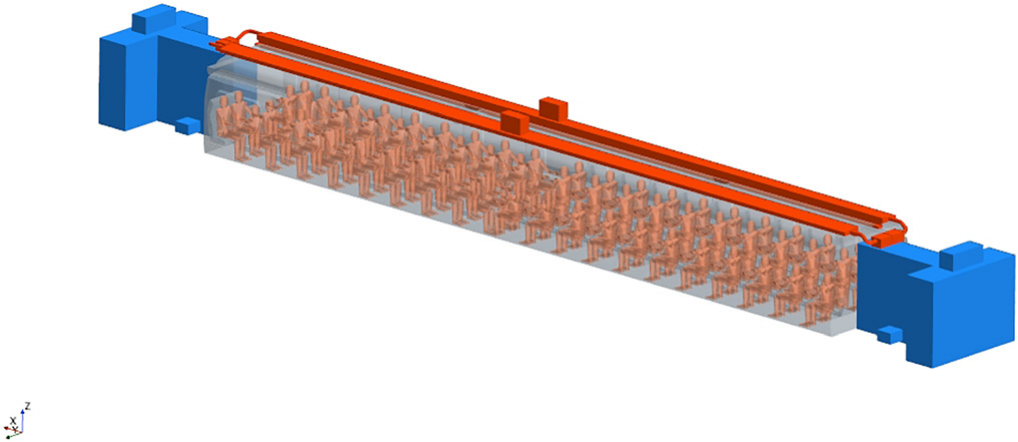
Computational domain representing the layout of a typical interregional train: ducts, central passenger compartment, and rear/front vestibules.

As shown in Fig. 14, the computational domain for the considered interregional train passenger compartment consists of four different regions. The air conditioner placed on the top of the passenger compartment treats air that enters the computational domain through red-colored ducts. The central portion represents the passenger compartment area in which up to 80 passengers can seat, while the two blue-colored extremities correspond to the front and back vestibules areas.

#### 1. Boundary conditions

The considered volumetric flow rate of treated air ($\dot{V}$) and its corresponding breakdown (with regard to the regions described before) is reported in Table VII. As already explained to a certain extent in Sec. IV B, only a critical situation is investigated; that is, typical winter conditions at low temperature and high relative humidity within the comfort zone indicated by EN13129 standard and reported in Fig. 13 (point B1, see also Table VIII). Moreover, the volumetric rate of flow recirculation and exhaust airflow in each vestibule is reported in Table IX.

**TABLE VII.** Volumetric flow rate ($\dot{V}$) of air treated by the HVAC system and its repartition into characteristic domain regions.

	$\dot{V}$	
	($m^{3}/h$)	(%)
Front vestibule	185	3
Rear vestibule	185	3
Passenger compartment	5570	94
Total	5940	100

**TABLE VIII.** Boundary conditions: air temperature and relative humidity (RH).

*T_air_*	23 °C
RH	65%

**TABLE IX.** Exhaust and recirculation volumetric flow rate ($\dot{V}$) in each vestibule. Outflow in each vestibule is 50% of the total treated volumetric flow rate.

	$\dot{V}$	
	($m^{3}/h$)	(%)
Recirculation	2438	42
Right extractor	266	4
Left extractor	266	4
Total outflow/vestibule	2970	50

#### 2. Grid sensitivity

**FIG. 15.**
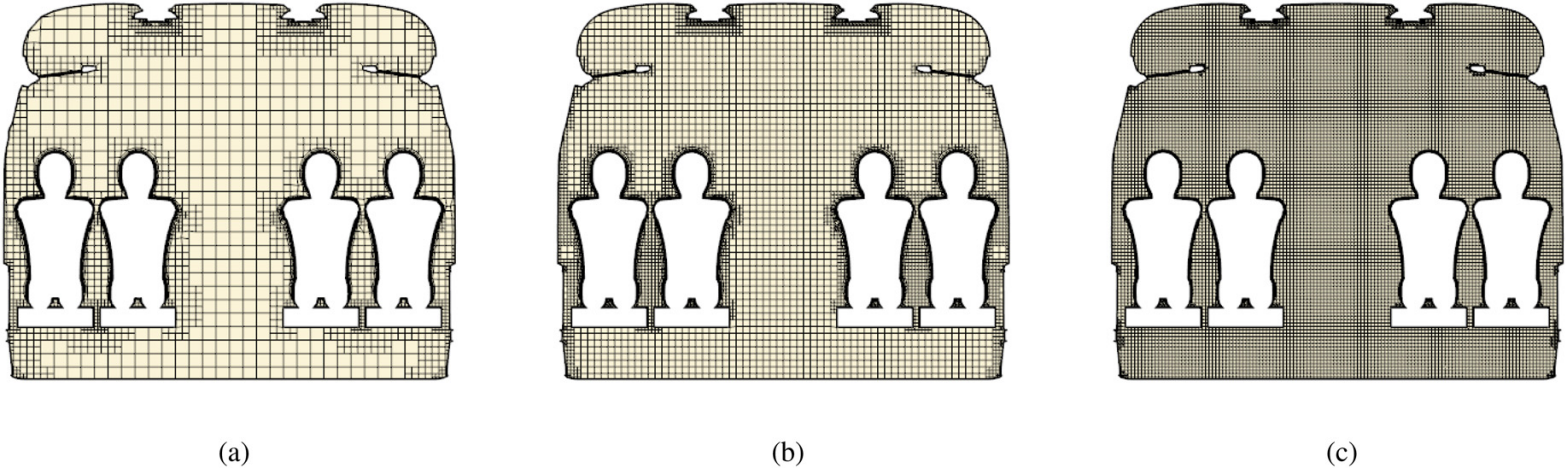
Coarse, medium, and fine mesh visualization at section plane A indicated in Fig. 16. (a) Coarse. (b) Medium. (c) Fine.

The grid for the computational domain has been chosen after the following considerations. Three different types of grids have been considered under the same boundary conditions defined in Sec. IV C 1. The characteristic size of such coarse, middle, and fine grids is indicated in Table X (see also Fig. 15).

**TABLE X.** Cell base size of coarse, middle, and fine grids (mesh sensitivity analysis).

	Unit	Coarse	Middle	Fine
Cell size	(mm)	80	40	20

**FIG. 16.**
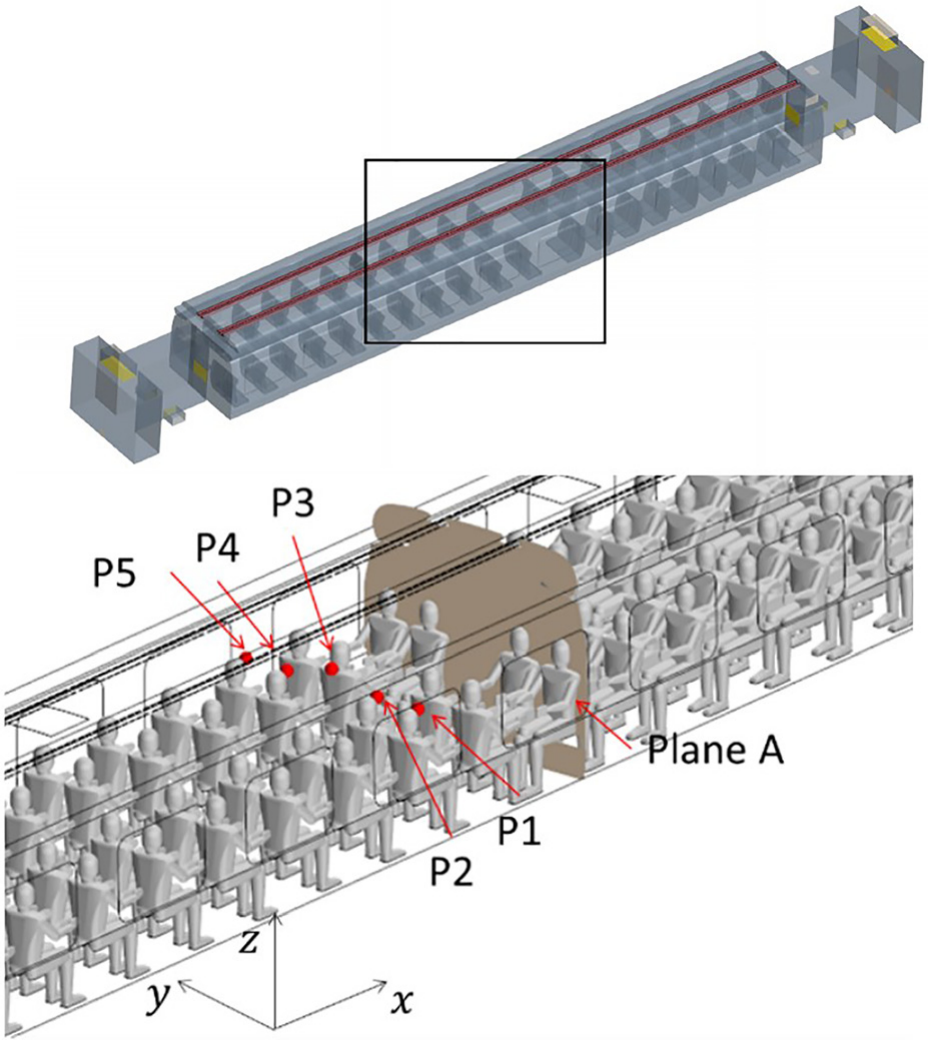
Probe locations and section plane used for the grid sensitivity analysis. Probe locations are indicated by points “P1, P2, P3, P4, P5” and plane section is Plane A.

The outcomes of such a comparative study are reported in terms of summation of the instantaneous velocity field on local cell faces, measured in the probe positions, indicated in Fig. 16 by red points P1, P2, P3, P4, and P5, and velocity field contour on the plane section A (see again Fig. 16).

**FIG. 17.**
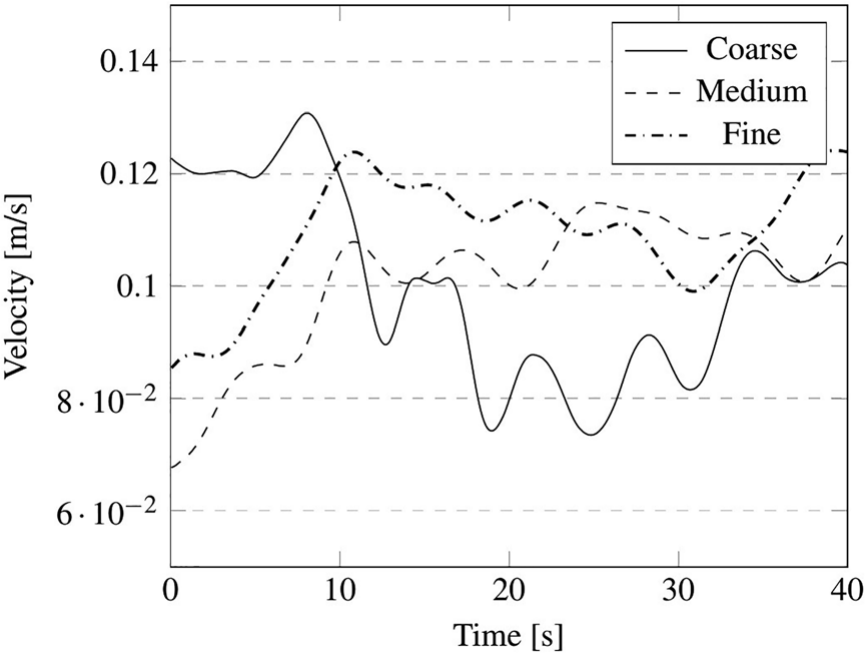
Summation of the instantaneous velocity field on cell face located at position of probe P1 in Fig. 16.

**FIG. 18.**
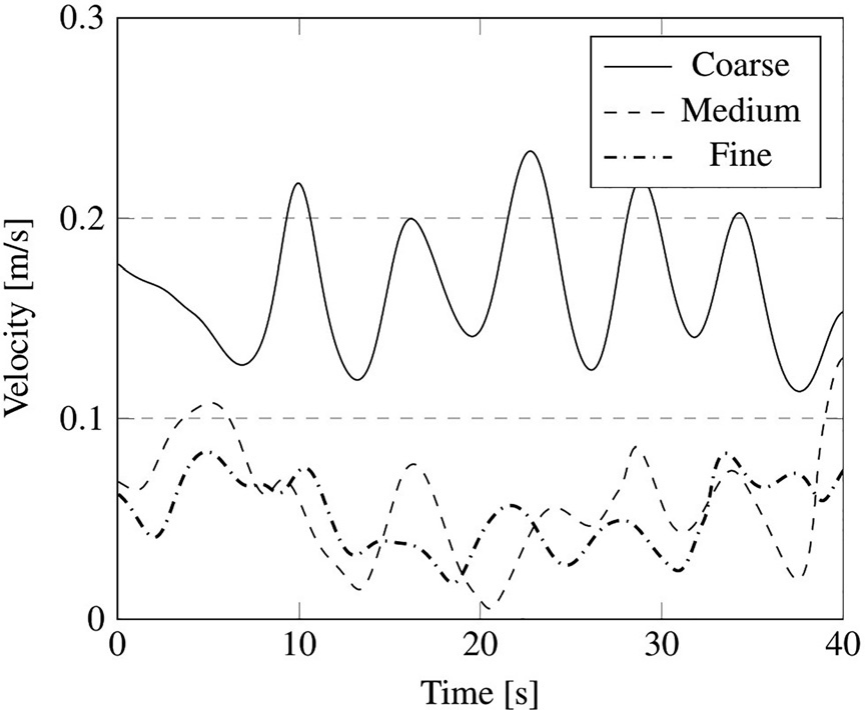
Summation of the instantaneous velocity field on cell face located at position of probe P2 in Fig. 16.

**FIG. 19.**
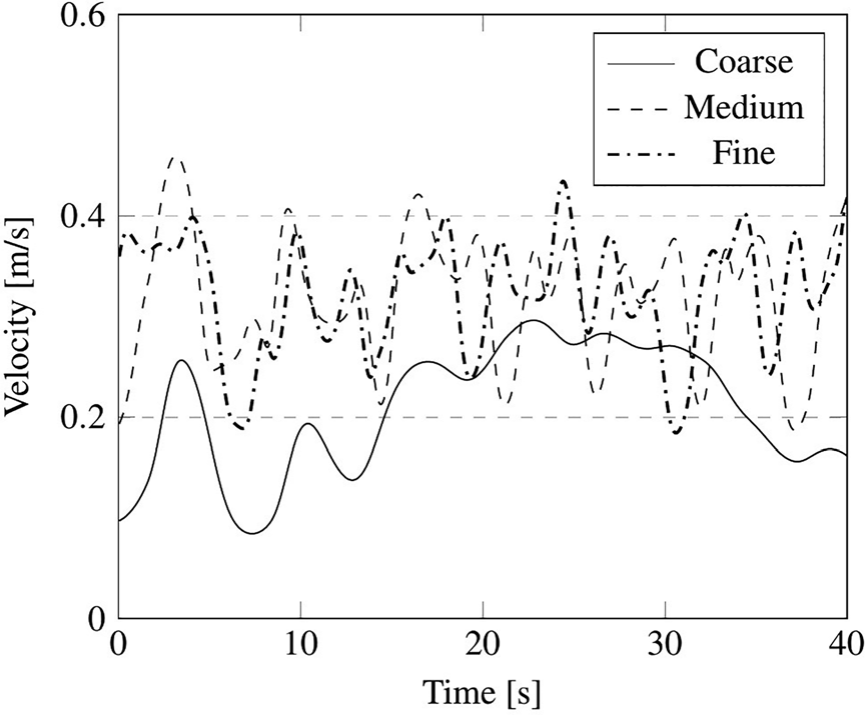
Summation of the instantaneous velocity field on cell face located at position of probe P3 in Fig. 16.

**FIG. 20.**
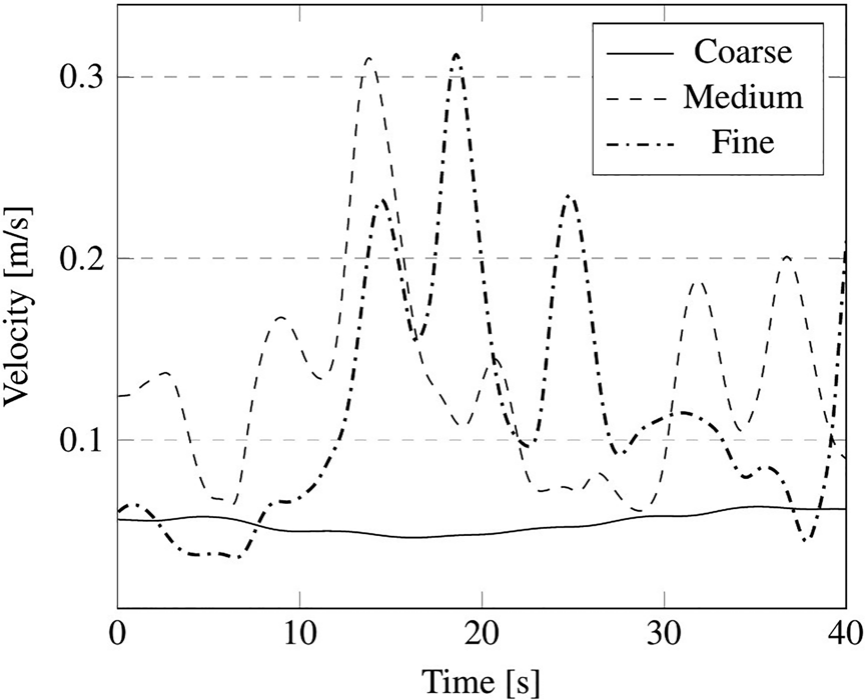
Summation of the instantaneous velocity field on cell face located at position of probe P4 in Fig. 16.

**FIG. 21.**
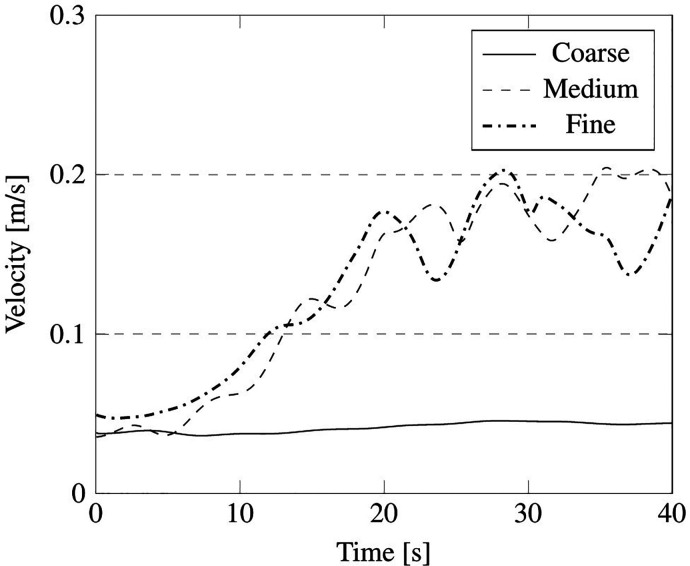
Summation of the instantaneous velocity field on cell face located at position of probe P5 in Fig. 16.

We wish to remark that, as considering the unsteady fluctuation of the velocity field is more relevant from the point of view of particle dynamics, the mesh sensitivity analysis has been based on this unsteady quantity. In particular, the aforementioned velocity summation has been recorded over a simulation time of 40 s for the just mentioned probes P1, P2, P3, P4, and P5 (the results have been reported in Figs. 17–21, respectively).

As qualitatively and quantitatively substantiated by these figures, the fluctuation of velocity in time is similar for the medium and fine grid in all the considered positions, in terms of both amplitude and frequency (with the exception of the coarse grid, an analogous distribution of peaks and valleys can be seen when the other two grids are used).

**FIG. 22.**
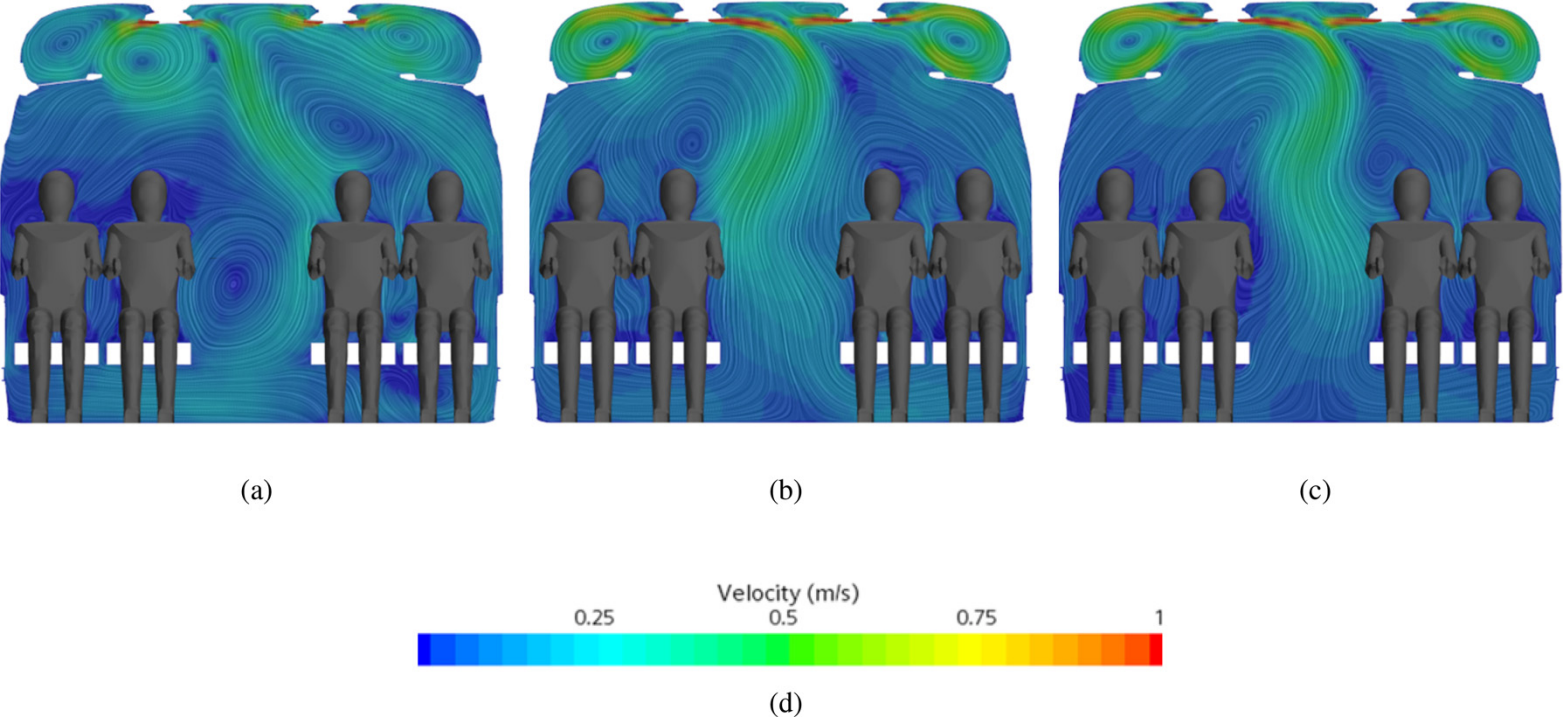
Mean velocity field at section plane A indicated in Fig. 16 obtained with coarse, medium, and fine mesh described in Table X. (a) Coarse. (b) Medium. (c) Fine. (d) Contour legend.

As a further demonstration of this outcome, Fig. 22 shows the mean velocity field in the plane A of Fig. 16. The significance of these figures resides in their ability to make evident that all the examined grids are able to capture the counter-rotating vortices in the upper part of the passenger cabin (in correspondence with the luggage compartment). It can be seen that the patterning behavior is essentially identical for the medium and fine grids, whereas a change in the symmetry of the main plume can be noticed when the coarse mesh is used.

On the basis of these results and related arguments, the middle mesh is therefore used for the simulations presented in Sec. IV D and IV E.

**FIG. 23.**
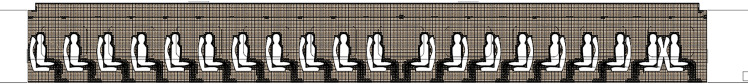
Selected mesh for droplet evolution case is the medium one.

In particular, the final mesh is reported in Fig. 23. As a concluding remark for this section, we wish to highlight that, in order to capture properly boundary-layer effects, a series of 10 prism layers has been implemented, with a first layer thickness such that the wall y+ function is in the acceptable range of [30:80].

### D. Droplet case study

Droplets are released according to the initial conditions reported in Tables XI and XII for coughing and talking cases, respectively. All the passengers are assumed to hold fixed positions, that is, to be static. Moreover, four different cases are analyzed, namely:

**TABLE XI.** Droplet initial conditions. Specified values for case A coughing passenger.

	Unit	Coughing
Cases		A
Injection time	(s)	0.1
Total mass	(mg)	24.6
Number	(/)	1900
Initial diameter	(*μ*m)	CDF^a^
Min diameter	(*μ*m)	1
Max diameter	(*μ*m)	2000
Cone angle	(deg)	19
Velocity	(m/s)	14.4
Temperature	(°C)	23

^a^ Cumulative distribution function during coughing, from Xie *et al.*^61^

**TABLE XII.** Droplet initial conditions. Specified values refer to each single talking passenger.

	Unit	Talking	
Cases		B-C-D	B
Injection time	(s)	0.1	5.0
Total mass	(/)	1.7 mg	1.7 mg/s
Number	(/)	1900	2000/s
Initial diameter	(*μ*m)	CDF^a^	
Min diameter	(*μ*m)	1	1
Max diameter	(*μ*m)	500	500
Cone angle	(deg)	24.2	24.2
Velocity	(m/s)	4.1	4.1
Temperature	(°C)	23	23

^a^ Cumulative distribution function during talking, from Xie *et al.*^61^

Case A—single passenger coughing;
Case B—single passenger talking for 0.1 and 5 s;
Case C—6 passengers talking;
Case D—10 passengers talking.

For each circumstance, the released number of particles is set according to the indications provided by Chao *et al.*^63^ and Stadnytskyi *et al.*^64^ Similarly, the droplet initial diameter has been defined according to the data reported in the experimental study conducted by Xie *et al.*^61^ where a tabular CDF (cumulative distribution function) was given for the initial diameter distribution of droplets in such situations. Other relevant experimental studies (Kwon *et al.*^65^) have shown that the cone angle of droplet ejection depends upon the gender of the person (the values used for this study are typical of the male gender).

For case B, two fundamental situations are considered, namely, B1 and B2. In the first, a talking passenger releases 1900 particles (corresponding to the amount of particles usually emitted in 1 s) “impulsively,” that is, in the same time that would be required to release them in a coughing event (0.1 s). In the second case, 2000 particles per second are released over 5 s. These situations obviously correspond to two limiting conditions, namely, a single short sentence such as “how are you?” and a more involved speech (including 5 or more complete sentences).

**FIG. 24.**
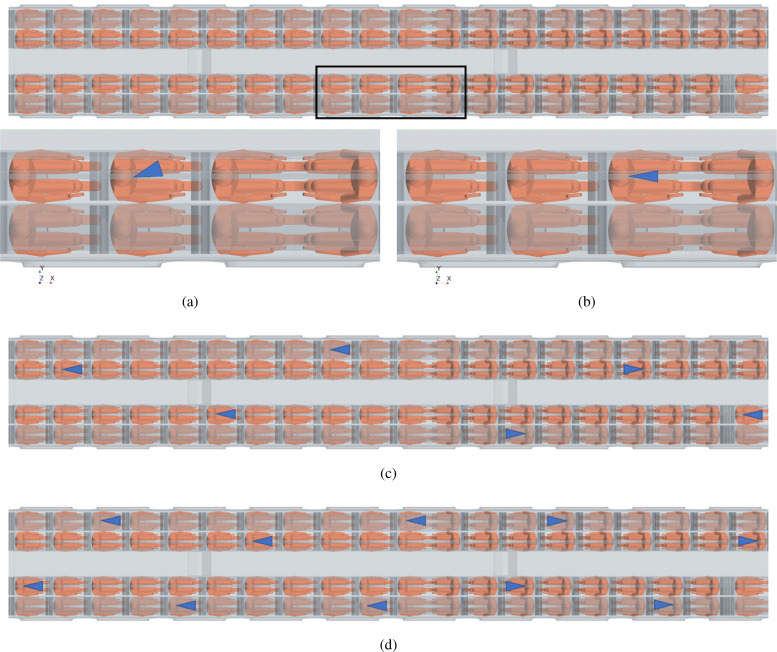
Cone injector position for Cases A, B, C, and D. Case A: cone injector axis is rotated of 20° and 10° respect to *xz* and *xy* plane, respectively. Cases B, C, and D: cone injector axis is parallel to x axis. (a) Case A—single passenger coughing. (b) Case B—single passenger talking. (c) Case C—6 passengers talking. (d) Case D—10 passengers talking.

Droplets are assumed to enter the domain through a cone injector whose position is illustrated in Fig. 24. Specifically, the cone injector axis of case A, Fig. 24(a), has a been rotated by 20° and 10° with respect to the *xz* and *xy* planes, respectively, and the cone injector of case B, Fig. 24(b), is parallel to the x axis, while for cases C and D, Figs. 24(c) and 24(d), we have considered 6 and 10 cone injectors, respectively, parallel to x axis and directed in the speaking direction of each passenger.

### E. Results

#### 1. Eulerian field—Steady state

**FIG. 25.**
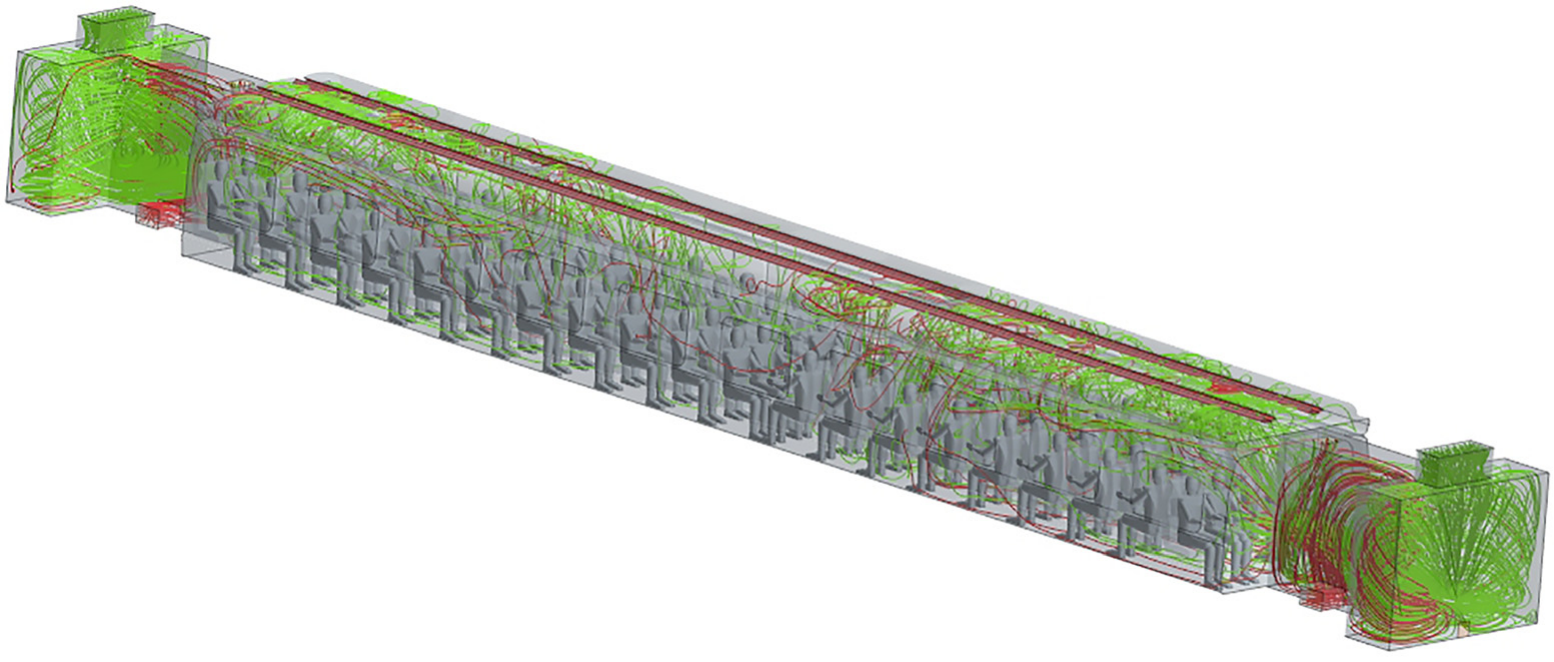
Streamlines in the cabin. All streamlines represent air coming from inlet ducts, green ones are collected by recirculation ducts in the front and rear vestibules, and red ones represent exhaust air expelled from outlets.

The outcomes of the numerical simulations in terms of Eulerian velocity field for the conditions defined in Sec. IV C 1 are reported in Fig. 25 in terms of streamlines.

As explained before, the air enters the passenger cabin from the ceiling ducts (blue streamlines in Fig. 25), each one consisting of two rows of ventilation inlets: an external one in correspondence with the luggage compartment and an internal one located on the aisle side.

It can be seen that the airflow spans the entire passenger compartment and reaches the grille opening. At this stage, mass flow is symmetrically partitioned into two streams: 50% going in the front and 50% in the rear vestibule, respectively.

**FIG. 26.**
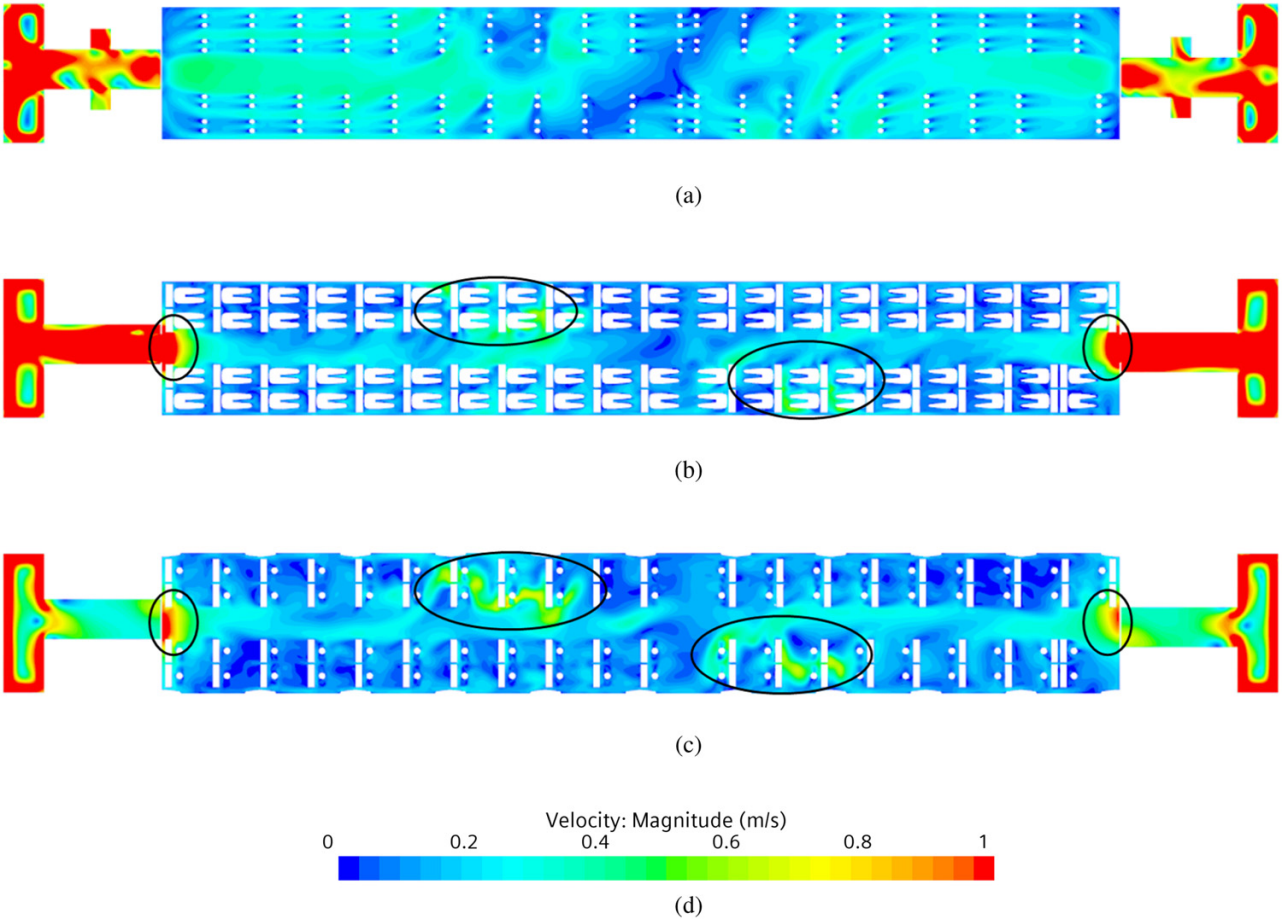
Mean velocity field on horizontal planes at different heights. (a) Horizontal plane at height *z *=* *0.1 m. (b) Horizontal plane at height *z *=* *0.6 m. (c) Horizontal plane at height *z *=* *1.1 m. (d) Contour legend.

As already explained to a certain extent in Sec. IV A, airflow is then split further into a recirculation flow (green streamlines in Fig. 25) and an exhaust (red streamlines in Fig. 25) current, the first is forced in the top recirculation duct, and the second is expelled through the side ducts near the floor. Some additional insights into the flow field can be gathered from Fig. 26. EN standard requirements prescribe a maximum value for the mean velocity as a function of mean cabin temperature, to be satisfied at specific passenger positions. Figure 26 shows the behavior at three distinct heights (horizontal planes): *z *=* *0.1, *z *=* *0.6, and *z *=* *1.1 m. For a cabin mean temperature of 23 °C, the maximum airspeed allowed is 0.4 m/s. As the reader will realize by inspecting this figure, on the horizontal plane at height *z *=* *0.1 m, the mean velocity field is under the target value with the two areas near the vestibules reaching the target, in correspondence of the aisle. On the other planes, at heights *z *=* *0.6 and *z *=* *1.1 m, respectively, there are some areas exceeding the maximum allowed value, indicated by black lines in Figs. 26(b) and 26(c). These simple observations lead to the conclusion that the considered HVAC system satisfies the EN standard requirements except for some localized areas (which is normal). As we will show in Subsections IV E 2–IV E 5, the effective topology of the flow and its magnitude can have an influence on the particle motion.

#### 2. Case A—Single passenger coughing

**FIG. 27.**
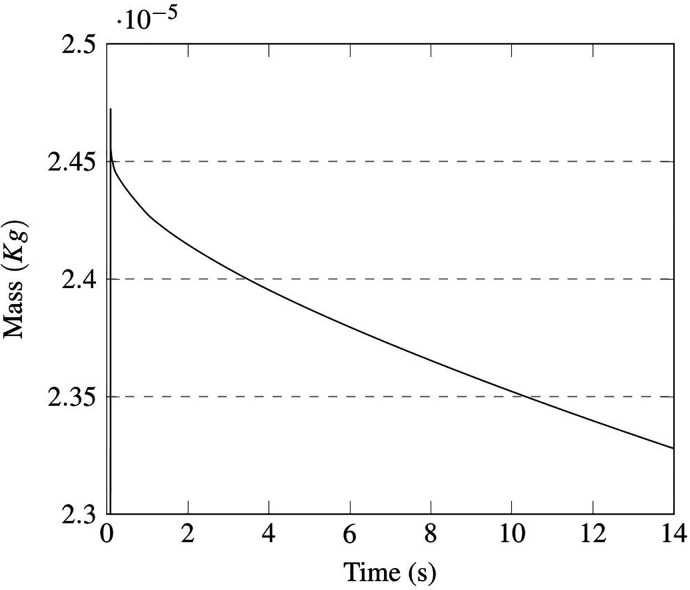
Case A—single passenger coughing. Time evolution of droplet mass.

**FIG. 28.**
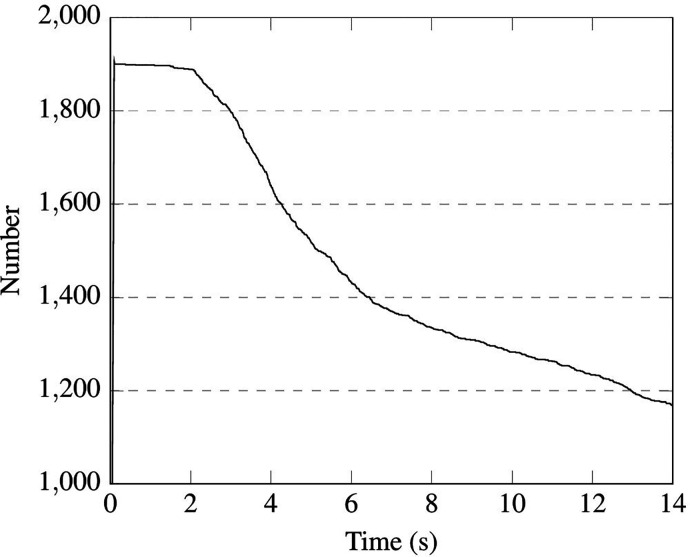
Case A—single passenger coughing. Time evolution of droplet number.

Here, we examine the coughing case. In particular, Figs. 27 and 28 account for the time evolution of the droplet total mass and number (this figure is instrumental in showing that the evaporation process starts as soon as particles are injected into the domain).

As a fleeting glimpse into this figure would confirm, in the first 2 s, the droplet evaporation rate is very high; this stage is followed by a new phase where the rate undergoes a shrinkage due to salt concentration effects. From a quantitative standpoint, in the first 6 s about 500 droplets evaporate, while other 200 droplets are lost in the last 8 s.

**FIG. 29.**
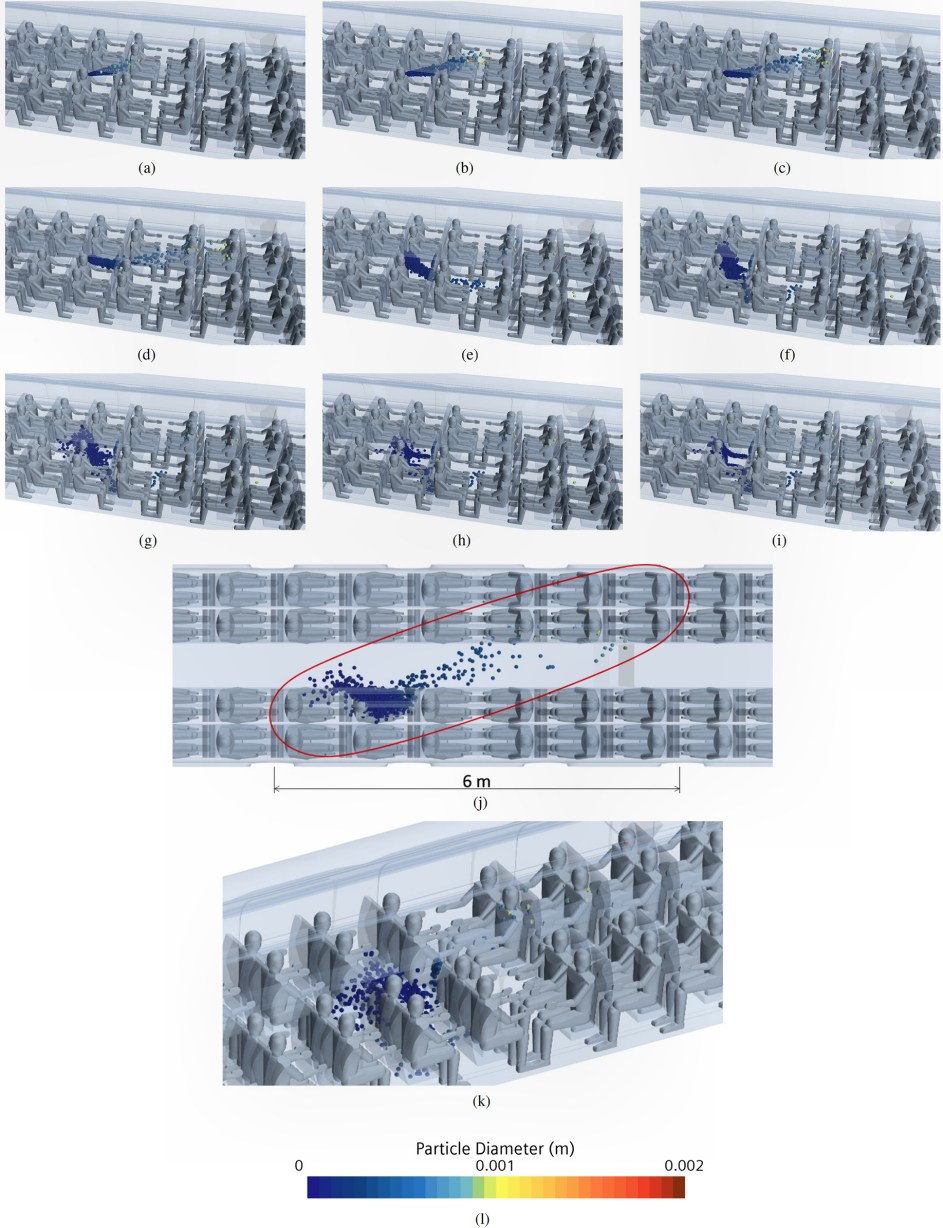
Case A—Coughing. Droplet diameter at different simulation times. (a) t = 0.1 s. (b) t = 0.2 s. (c) t = 0.3 s. (d) t = 0.5 s. (e) t = 1 s. (f) t = 2 s. (g) t = 5 s. (h) t = 10 s. (i) t = 14 s. (j) Maximum diffusion time (t = 5 s), top view. (k) Maximum diffusion time (t = 5 s), side view. (l) Contour legend.

These data are complemented by Fig. 29, where we have reported the droplet diameter contour at different times. Interestingly, up to a simulation time of t = 0.5 s, the ejected particle maintains the cone shape, the bigger particles being the ones that travel farther. After some time, the cloud of particles starts to feel the surrounding Eulerian (cabin) velocity field and, accordingly, undergoes some deformation. In particular, the transverse size of the cloud is maximized for t = 5 s [maximum spatial diffusion time, see the top and side views in Figs. 29(j) and 29(k), respectively].

Such figures are particularly useful as they can be used to get quantitative information on the particle spreading process (covering 6 m and affecting 6 rows of seats). For this case (with the hypotheses defined in Sec. IV D), the droplets would hit directly only 7 passengers.

#### 3. Case B—Single passenger talking

The companion (talking) case is depicted in the present section. As explained before, for this case two different scenarios have been simulated (see also Table XII):

Case B1—1900 droplets ejected impulsively (short sentence such as “hello guys” or “how are you?”);
Case B2—2000 droplets per second ejected over 5 s (continuous speech including several complete sentences).

**FIG. 30.**
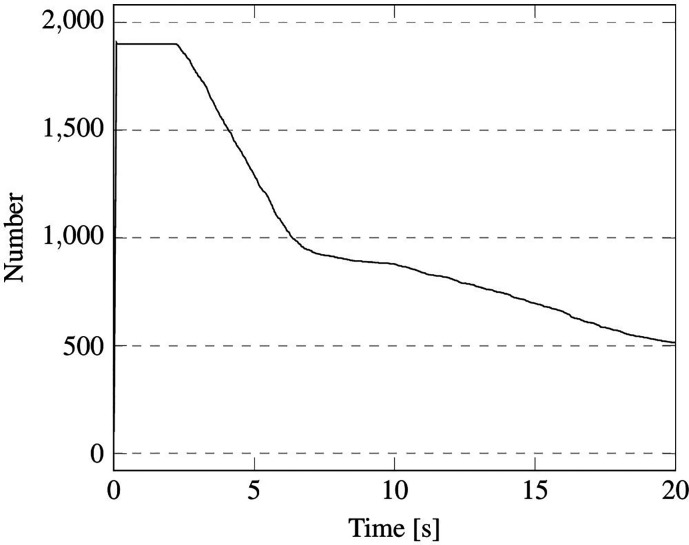
Case B1—1900 droplets injected in 0.1 s.

**FIG. 31.**
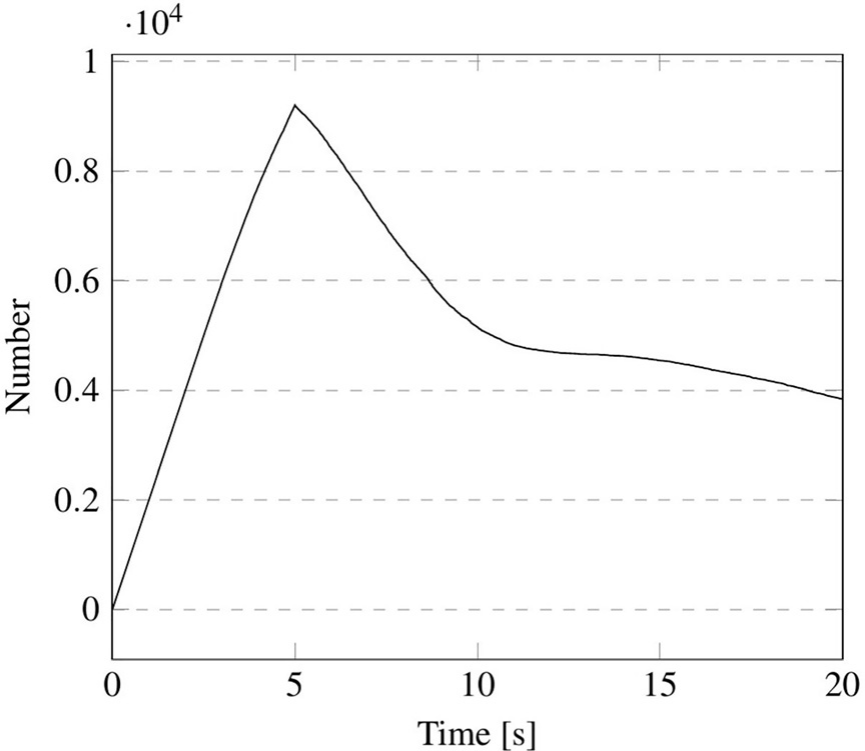
Case B2—2000 droplets/s injected in 5.0 s.

The time evolution of droplets number is reported in Figs. 30 and 31 for cases B1 and B2, respectively. It is worth noticing that for the latter case the total number of particles never achieves the expected value of 10 000 due to the evaporation process, which (as time passes) causes some droplets (the smaller ones) to disappear quickly. Notably, in both situations, a rapid decrease in the droplet number can be seen just after the completion of the injection (droplet release) phase. This is yet due to the evaporation of the droplets with smaller size. After this stage (which lasts approximately 5 or 6 s regardless of whether a short sentence or continuous speech is considered), a less steep branch is obtained, which physically corresponds to the timeframe in which slower evaporation of bigger droplets occurs.

**FIG. 32.**
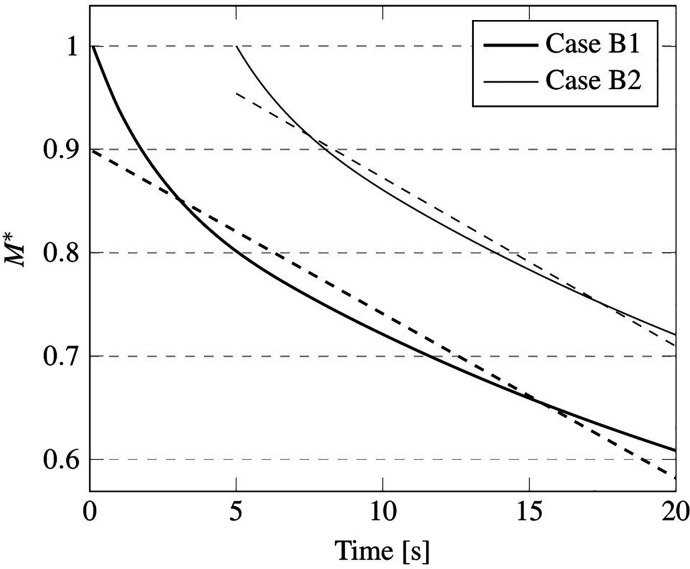
Case B—Non-dimensional mass $M^{*}$ and related linearly extrapolated evolution laws.

Interestingly, as evident in Fig. 32, if the non-dimensional mass is reported as a function of time, the decrease rate (i.e., the angular coefficient of the straight lines mimicking the quasi linear behavior of the considered trend) is independent from the injected mass (see Table XIII).

**TABLE XIII.** Non-dimensional mass $M^{*}$ decreasing rate for cases B1 and B2.

	B1	B2
dM^*^/*dt*	−0.016	−0.016

**FIG. 33.**
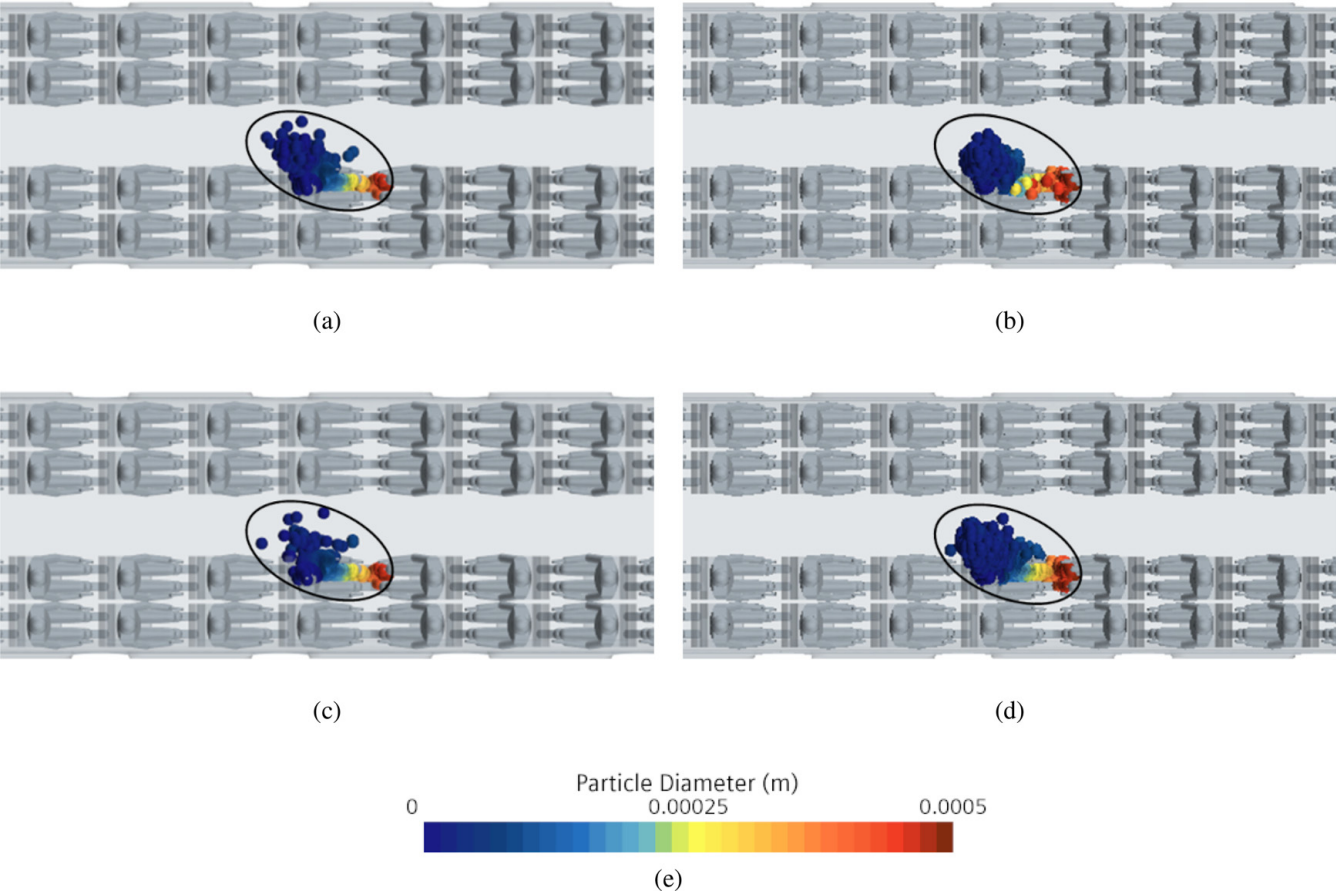
Case B—Talking. Comparison of droplet diameter at different simulation times for B1 and B2 cases. (a) Case B1 t = 5 s. (b) Case B2 t = 5 s. (c) Case B1 t = 7.5 s. (d) Case B2 t = 7.5 s. (e) Contour legend.

An explanation/justification for this finding can be elaborated in its simplest form on the basis of the argument that, regardless of the number of droplets being released in the cabin (1900 and 10 000 for cases B1 and B2, respectively), the amount of water evaporating is so small (in terms of volume fraction) that it is not able to change the air humidity content appreciably [on which the evaporation rate depends, as mathematically expressed by Eq. (25)]. Another remarkable implication of this observation is that the maximum distance the droplets can travel away from the source emitting them does not depend on their number (as substantiated by the 3D views in Fig. 33); rather, it is a function of the droplet diameter (distribution) only and of the ambient temperature.

This is the reason for which cases C and D being discussed in Secs. IV E 4 and IV E 5, respectively, are simulated considering the impulsive injection condition (with 1900 droplets) only (this condition leading to notable computational savings and being sufficient to evaluate the maximum possible extension attained by the cloud).

Before moving to the cases with multiple sources, however, meaningful insights also follow from a comparison of Fig. 33 with the analogous ones for the coughing testbed Fig. 29.

Although the behaviors of particle total mass and number are relatively similar in terms of trends and amplitudes, for the talking case, the droplet evolution in space is seemingly much more limited and, accordingly, the passenger exposition risk is lower.

Notable differences can also be identified in the morphology of the cloud shape and the related spatiotemporal evolution.

**FIG. 34.**
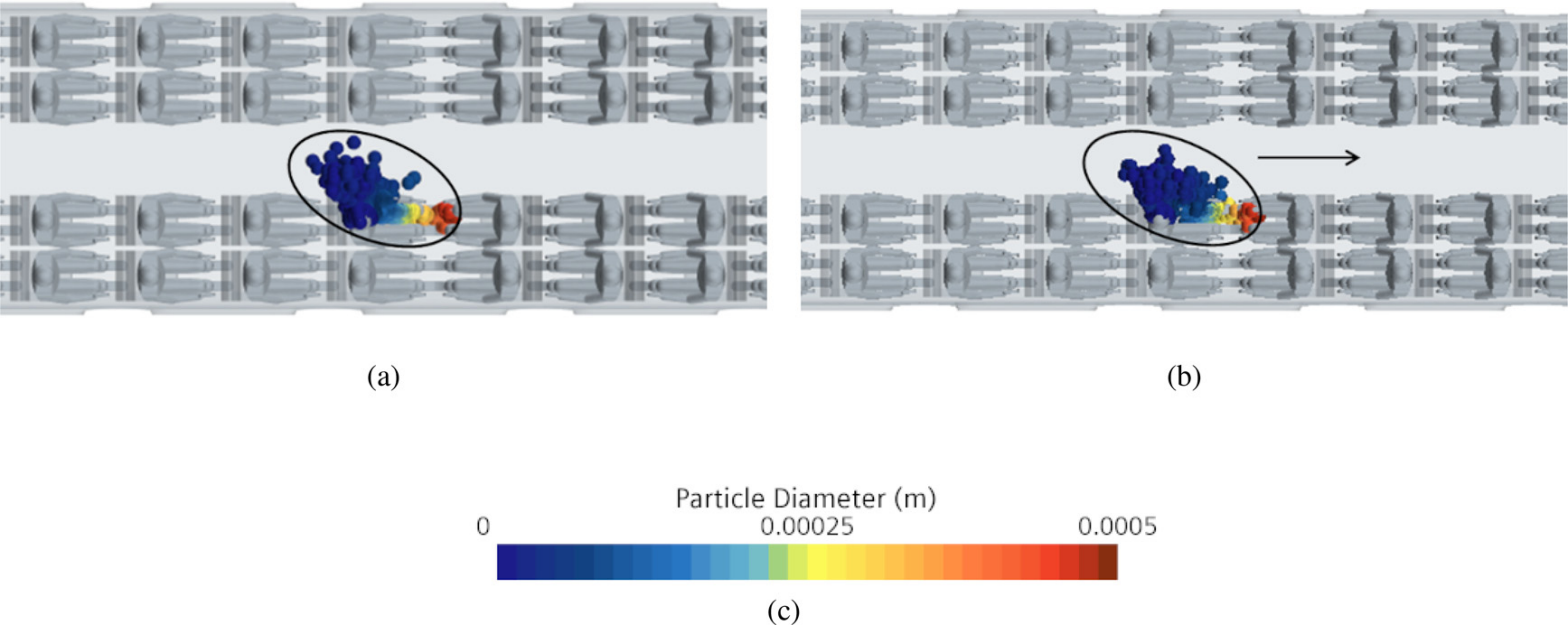
Case B—Talking. Snapshot at t = 5 s for B1 case. (a) Normal scenario. (b) Emergency braking scenario (the arrow indicates the motion direction). (c) Contour legend.

As a concluding remark for this section, we wish to report that, for the sake of completeness, we have also simulated the case in which the train undergoes a variation of velocity along its main direction of motion (i.e., $a_{x}\neq 0$, *a_y_* = 0, all the cases presented previously being obtained for $a_{x}=a_{y}=0,\;a_{z}=-g$). More precisely, we have examined the interesting scenario corresponding to an emergency braking of the considered interregional train (*V_max_* = 160 km/h). For such a situation, a typical deceleration value is of the order of 1.3 ${m/s}^{2}$. In order to mimic these circumstances, $a_{x}=-1.3\;\;{m/s}^{2}$ has been considered in the governing equations in addition to *a_y_* = 0 and $a_{z}=-g$ and the case B1 has been simulated one more time. The related results, along with the companion case B1 without deceleration, are reported in Fig. 34. As witnessed by this figure, the effect of the considered inertial force can be considered almost negligible, which explains why the remaining cases (see Secs. IV E 4 and IV E 5) have been yet simulated assuming that the train moves at a constant speed.

#### 4. Case C—6 passengers talking

**FIG. 35.**
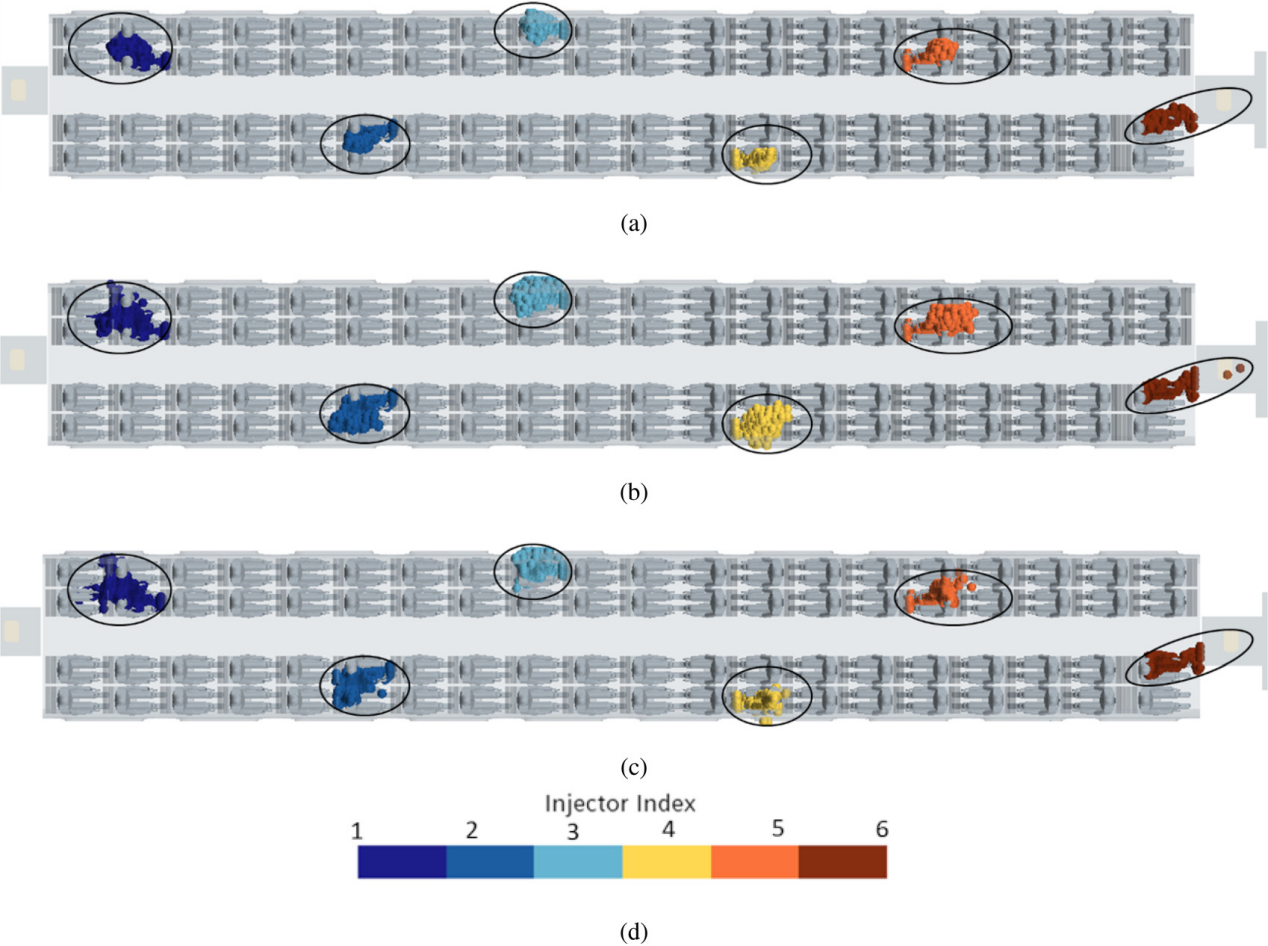
Case C—6 passengers talking. Time evolution of droplets for different talking passenger positions. (a) t = 2 s. (b) t = 5 s. (c) t = 14 s. (d) Contour legend.

A more involved scenario is considered in this subsection where the number of talking heads is increased to six [uniformly distributed in the cabin as shown in Fig. 24(c)]. The corresponding time evolution of the droplets is reported in Fig. 35 at different simulation times; in this figure, we have also indicated the possible “area of influence” related to each talking passenger, clearly showing that 6 talking passengers may infect up to 9 passengers (in a statistical sense, 7.5% of the passengers may infect up to 11% of the remaining passengers).

#### 5. Case D—10 passengers talking

In view of results reported in the above two subsections, here we discuss the even more realistic scenario in which a series of 10 talking passengers are disposed (evenly spaced) as shown in Fig. 24(d). The total mass and number of droplets are 10 times the corresponding values for the single talking passenger case; moreover, the related temporal trends (not shown) are rather similar to those already discussed for case B1.

**FIG. 36.**
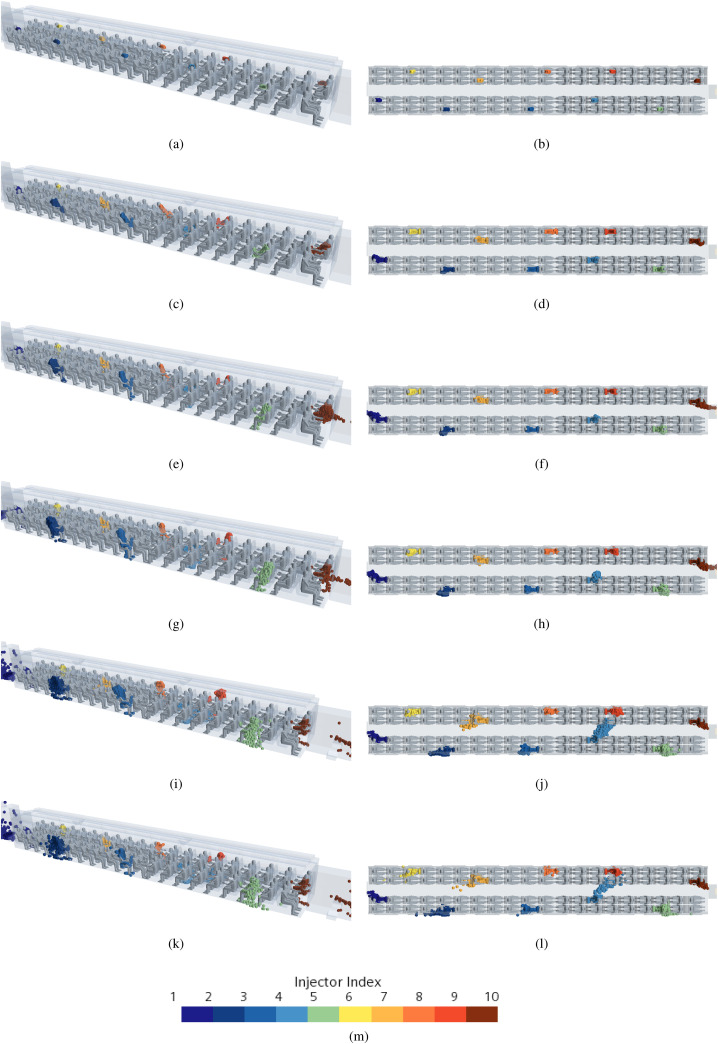
Case D—10 passengers talking. Time evolution of droplets for different talking passenger positions. (a) t = 0.1 s (diagonal view). (b) t = 0.1 s (top view). (c) t = 0.5 s (diagonal view). (d) t = 0.5 s (top view). (e) t = 1 s (diagonal view). (f) t = 1 s (top view). (g) t = 2 s (diagonal view). (h) t = 2 s (top view). (i) t = 5 s (diagonal view). (j) t = 5 s (top view). (k) t = 14 s (diagonal view). (l) t = 14 s (top view). (m) Contour legend.

Figure 36 shows the time evolution of the droplets, specifically each different color is representative of a specific position of the considered talking passenger. In this case (for the sake of brevity), only a global view of the complete scenario inside the train cabin is reported (diagonal and top views only).

In line with the findings already illustrated in Subsections IV E 2 and IV E 3, these results confirm that for a talking passenger near the centerline of the train cabin, the maximum droplet diffusion area (or “generated risk” area) is attained after 5 s after the emission of the droplets. Interestingly, however, a completely different situation can be noticed with regard to the passengers located near the vestibules and at intermediate positions.

**FIG. 37.**
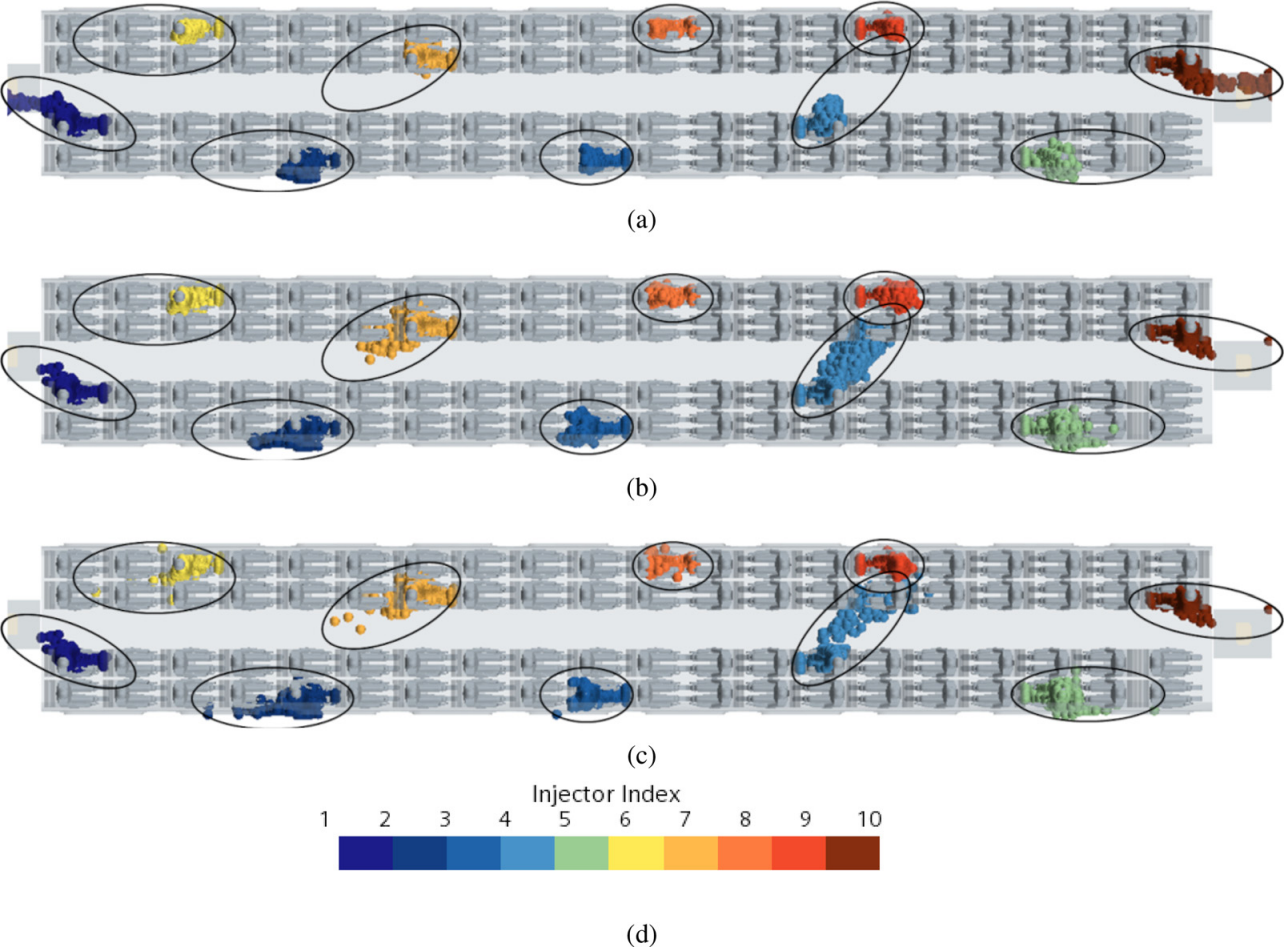
Case D—10 passengers talking. Time evolution of droplets for different talking passengers' positions. (a) t = 2 s. (b) t = 5 s. (c) t = 14 s. (d) Contour legend.

This can be appreciated in Fig. 37 where dark lines are used to highlight the aforementioned “areas of influence.” In particular, for the passengers near the vestibules, the cloud shape (or risk region) tends to be stretched toward the vestibules following the flow going in that direction. As a result of the acceleration undergone by the air (as it moves toward the vestibules), the maximum extension of the cloud of particles is no longer attained after t = 5 s (rather it changes with time continuously).

**FIG. 38.**
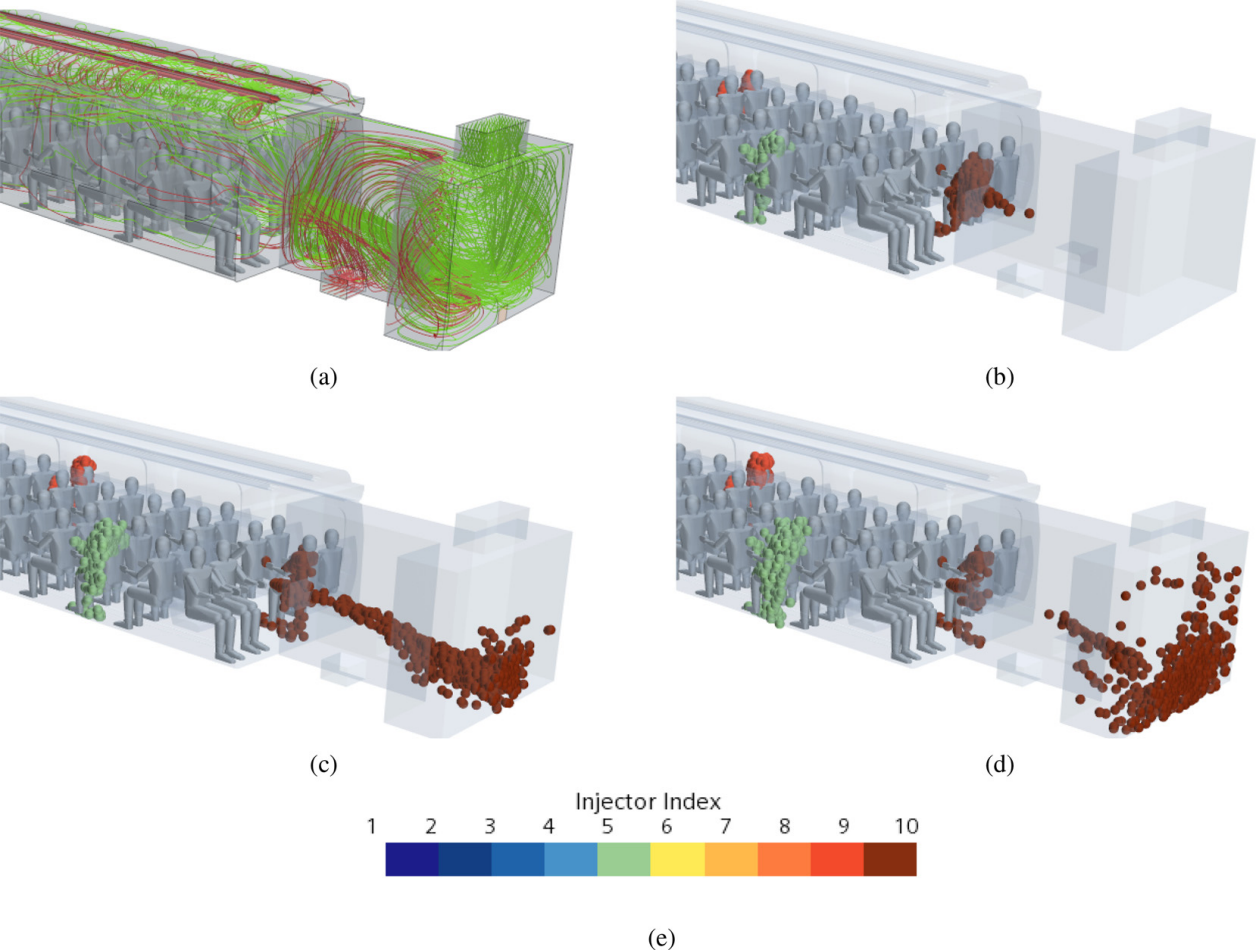
Case D—10 passengers talking. Droplet evolution in front of the vestibule area. (a) Streamlines representing exhaust (red) and recirculation (green) airflow. (b) t = 1 s. (c) t = 2 s. (d) t = 3 s. (e) Contour legend.

Other meaningful information, following natural from an inspection of Fig. 38, concerns the evident relatively high risk, which passenger temporarily located inside the vestibules would be exposed to. In those regions, the percentage of recirculating flow is relatively high (42% of total volumetric flow rate, as reported in Table IX). As a matter of fact, particles released from nearby passengers reach these areas in just a few seconds.

As a concluding remark, it is worth highlighting that this simulation also provides potentially useful statistical data (to be considered together with those already obtained for the 6-talking-heads case). Ten passengers (evenly spaced inside the cabin) talking without a face mask can produce droplets able to hit about 20 passengers (12% of passengers could theoretically infect up to 25% of the remaining cabin occupants).

## V. CONCLUSIONS

The diffusion of the COVID-19 pandemic depends on a multitude of influential factors, many of which are of a purely thermo-dynamic or fluid-dynamic nature. These, in turn, require different levels of analysis, which range from the study of the intrinsic (fundamental) physical mechanisms by which nature operates to more practical aspects and the specific intricacies connected with the complexity of the environment where human beings leave and operate.

In the present study, the dispersion of evaporating saliva droplets into a train cabin has been analyzed from both the traditional coarse-grained Eulerian (i.e., continuum) perspective and from a fine-grained micromechanical level in which all the saliva droplets have been tracked individually together with the related content of water, momentum, and energy. Special care has been devoted to the description of the required mathematical models and numerical methods, with the explicit intent to create a theoretical framework on which other future studies may rely. In doing so, the state of the art has been considered in terms of existing paradigms that have already proven to successfully deal with most (if not all) of the above-mentioned influential factors. Following experimental studies on the subject, dilute dispersions of multi-dispersed droplets have been considered. On the one hand, the multi-dispersed nature of these distributions has allowed the implementation of realistic settings, where the proper differences (in terms of droplet number and distribution) affecting talking and coughing events have been taken into account. On the other hand, the dilute nature of the considered multi-phase flow has represented the necessary pre-requisite for the application of a two-way coupling strategy (thereby alleviating us from the burden to account for particle–particle interactions in the frame of four-way coupled or similar numerical approaches). The evaporating nature of the droplets has been also modeled, taking into account both concentration and temperature gradients. Moreover, the formation of solid nuclei due to the crystallization of salt dissolved in the droplets has also been considered, as this can yet have a non-negligible influence on the evaporation process. The high-fidelity representation of boundaries has not been limited to the environment, but has been applied also to the involved human beings (modeled as static mannequins). A part of our meticulous description of such methodological aspects has also resulted from the realization that existing studies are rare and sparse, which has hindered to a certain extent the development of general criteria and consensus about the most relevant approach for the analysis of this class of problems. In the majority of cases, these can be considered weakly compressible and can be therefore treated in the frame of pressure-based solvers and related extensions to low-Mach-number flows.

To make the outcomes of such a study as much realistic as possible, all the intricacies (in terms of the forced topology of the flow and morphology of the boundaries limiting it) have been considered and implemented. This has required *a priori* detailed analysis of the HVAC (heating, ventilation, and air conditioning) system for interregional trains, the effective standards used by engineers to design such systems and of the typical droplet emission processes associated with human beings. Moreover, fully three-dimensional (extremely demanding) numerical simulations have been used to adequately account for all these aspects.

This framework has led us to identify the average behavior of particles by revealing their spatiotemporal evolution. We have connected such statistics to the evaporation process, giving deeper insights into the particle transport mechanisms in conjunction with the considered complex flow topology and typical physiological or natural events such as talking and coughing.

It has been shown that with all the relevant effects taken into account, the paths of the incompressible liquid particles are intertwined and connected with the intricacies of the flow established inside the train cabin as a result of the air conditioning system. Nevertheless, the initial conditions (nature of the considered physiological event) have also a remarkable impact on the droplet cloud evolution in terms of symmetry and droplet transport rate, which finally result in a different risk level for the occupants of the cabin.

In a nutshell, the main outcomes of the present numerical study can be summarized as follows.

A single coughing passenger seating in proximity to the compartment centerline can produce droplets potentially impinging on up to 7 distinct human beings. In this specific case, particles are faster than those released in an equivalent talking event. Therefore, as expected, a coughing event leads to a more dangerous scenario.

More realistic circumstances, however, are represented by the situation in which a relatively high number of passengers are talking. In order to obtain statistically meaningful data for these cases, we have considered talking mannequins periodically positioned along with the entire extension of the compartment. The main outcomes of the related simulations are summarized in Table XIV.

**TABLE XIV.** Number of potentially infecting passengers and corresponding number of cabin occupants exposed to the risk of infection.

Infecting	Infected
Coughing	
1	7
Talking	
1	1
6	9
10	20

Some additional insights stem naturally from Table XV where we have collected specific data extracted from the case with 10 talking passengers (Fig. 37). Under a slightly different perspective, the companion Table XVI indicates the maximum, minimum, and mean number of non-talking passengers exposed to the risk of infection as a function of a “discrete subset” *R* of talking heads (the variation in the numbers being due to the specific talking passengers considered as source of droplets).

**TABLE XV.** Passengers exposed to droplet clouds for each passenger injector index according to Fig. 37.

Injector index	Passengers exposed to droplets
1	0
2	3
3	1
4	5
5	3
6	4
7	2
8	1
9	1
10	0

**TABLE XVI.** Minimum, maximum, and mean number of passengers exposed at risk considering different combinations of talking passengers (*R*) inside the train cabin.

*R*	No. of infected passengers		
	Min	Max	Mean
1	0	5	2.5
2	0	9	4.5
3	1	12	6.5
4	2	15	8.5
5	3	17	10

**FIG. 39.**
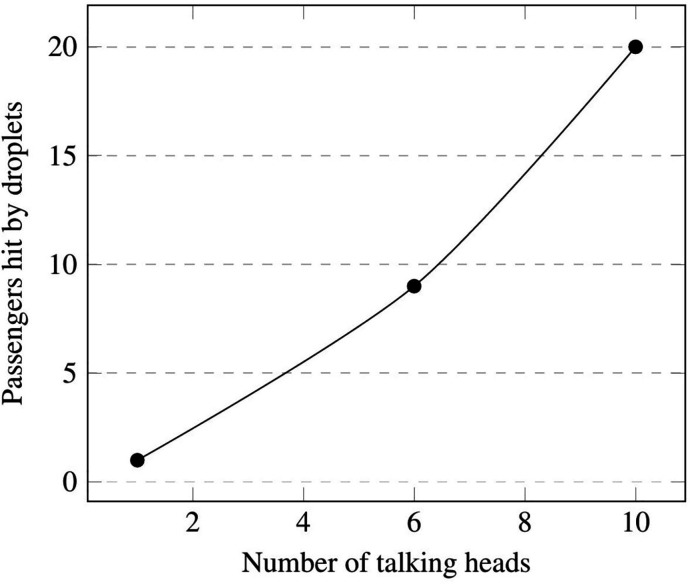
Trend line of passengers exposed to the risk of infection (hit by droplets) vs potentially infecting passengers (talking heads) according to investigated scenarios (cases B C D).

Finally, Fig. 39 shows the relationship between the number of passengers “at risk” (hit by droplets) and the number of droplet emitting heads. The significance of this final figure lies essentially in the evidence it provides about the quasi-linear relationship between the considered quantities and the related ratio (which we loosely define as the “train cabin infection spreading ratio” *η*). As the reader will realize by inspecting this figure, for the considered conditions *η* takes a value ≈2 (obviously, this value being valid only in the considered range of the talking heads number; a decrease in *η* should be expected due to saturation effects as the number of talking passengers increases).

Another interesting way to think about the present results is to consider that these simulations have revealed that if evaporation is properly taken into account, the size of the droplet clouds attains an asymptotic (maximum) value that does not depend on the time during which the talking events occur. This is because droplets evaporate completely as a certain threshold distance from the source is exceeded. Despite this “limitation” affecting the droplet lifetime, the droplet clouds can interfere with other passengers and lead to an intricate matrix of cause-and-effect relationships, as shown in Tables XV and XVI.

As a concluding remark, we wish to point out that, although it has been applied to a train cabin, this model could be used to track the particle spreading process in all those circumstances where some flow control is implemented (through air conditioning) and the Reynolds numbers take values comparable to that considered here, for example, the cabin of airplanes, offices, and restaurants.

**FIG. 40.**
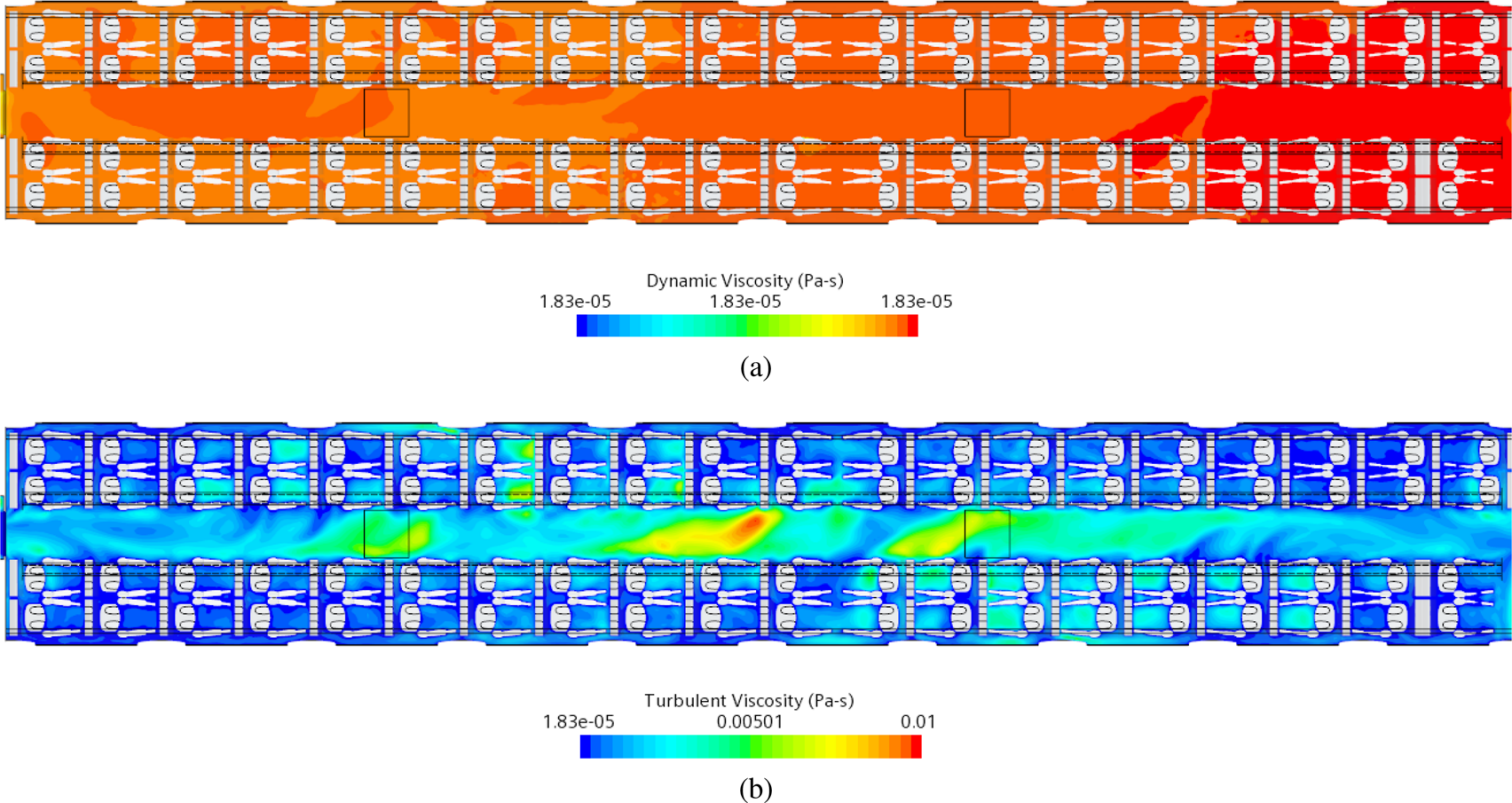
Viscosity distribution. (a) Dynamic viscosity. (b) Turbulent viscosity.

For the sake of completeness (and in order to get the additional insights into these processes), we have finally assessed the relative importance of molecular and turbulent diffusion through comparison of relevant contour maps showing the spatial distribution of the kinematic (molecular) viscosity (satisfying the well-known Sutherland's law for gases) and the turbulent viscosity. This has revealed that turbulent diffusion is dominant (the turbulent viscosity being three orders of magnitude larger than the molecular counterpart, see Fig. 40). A similar concept can also be applied to the other transport properties (thermal diffusivity and species diffusion coefficient) as both the molecular and turbulent Prandtl and Schmidt numbers take (approximately) unit values in the present work.

An exciting prospect for the future is to extend such analysis to non-nominal situations such as those resulting from an anomaly in the conditioning system. Moreover, more sophisticated evaporation models shall be elaborated that can account for the presence of mucus in the saliva droplets (especially with regard to coughing events).

## Data Availability

The data that support the findings of this study are available from the corresponding author upon reasonable request.
